# From complex data to clear insights: visualizing molecular dynamics trajectories

**DOI:** 10.3389/fbinf.2024.1356659

**Published:** 2024-04-11

**Authors:** Hayet Belghit, Mariano Spivak, Manuel Dauchez, Marc Baaden, Jessica Jonquet-Prevoteau

**Affiliations:** ^1^ Université de Reims Champagne-Ardenne, CNRS, MEDYC, Reims, France; ^2^ Université Paris Cité, CNRS, Laboratoire de Biochimie Théorique, Paris, France

**Keywords:** molecular dynamics, computed simulations, scientific visualization, computer graphics, data visualization

## Abstract

Advances in simulations, combined with technological developments in high-performance computing, have made it possible to produce a physically accurate dynamic representation of complex biological systems involving millions to billions of atoms over increasingly long simulation times. The analysis of these computed simulations is crucial, involving the interpretation of structural and dynamic data to gain insights into the underlying biological processes. However, this analysis becomes increasingly challenging due to the complexity of the generated systems with a large number of individual runs, ranging from hundreds to thousands of trajectories. This massive increase in raw simulation data creates additional processing and visualization challenges. Effective visualization techniques play a vital role in facilitating the analysis and interpretation of molecular dynamics simulations. In this paper, we focus mainly on the techniques and tools that can be used for visualization of molecular dynamics simulations, among which we highlight the few approaches used specifically for this purpose, discussing their advantages and limitations, and addressing the future challenges of molecular dynamics visualization.

## 1 Introduction

Molecular dynamics (MD) simulation (Battimelli et al., 2020) is an essential numerical method for understanding the physical basis of the structures, functions, and dynamics of biological macromolecules. It has been applied to biological systems for a few decades and was recognized in 2013 by the Nobel prize of chemistry. It consists of computational algorithms to calculate the time-dependent behaviour of a molecular system, providing detailed information on the fluctuations and conformational changes of macromolecules such as proteins and nucleic acids as well as many other classes of chemical compounds. Today, with the increase of High-Performance Computing (HPC) capabilities, it is possible to simulate the molecular behavior of very large molecular architectures for a longer duration, in some cases close to the time scales of experimental observations. The challenge now is to validate and analyze these simulations and scrutinize the corresponding associated trajectories to extract the biologically important information relating to the structures, the functions, and the dynamics of the biomolecules. Consequently, the visualization of these simulations is an essential tool to understand and interpret the dynamics of the simulated biological systems. It is also a good means to communicate scientific information (Lyall et al., 2023). In this sense, our report on the state of the art discusses various graphical representations. In addition, many software packages allow visualization of simulations through a variety of representations. However, the assessment of complex systems remains difficult with the classical repertoire of representations. Therefore, it is necessary to develop new visualization techniques and representations to facilitate the analysis and perception of relevant interactions. An important challenge today is to propose new approaches to visualize molecular simulations that adapt to the amount of data. In this review, we take stock of advances in visualization from MD. We outline the evolution of visualization techniques over time in the context of molecular dynamics simulations and highlight their limitations. We then present the challenges and prospects for further developments of molecular visualization techniques.

## 2 Methodology

In our methodology, we first defined the scope of this review. We decided to focus on techniques and approaches for visualizing molecular dynamics simulations, with particular emphasis on complex biomolecular systems. Developments in high-performance computing have given these numerical simulations a great boost. Simulation trajectories are being created for increasingly complex systems over longer and longer time periods. Scientific visualization is a crucial element of data analysis and interpretation. Our aim was to identify the tools and techniques available in the literature for visualizing such systems.

We started from a corpus of publications that we had already collected over the years, as our main activity is closely related to the scope of the review. In addition, we performed several systematic literature searches, in particular based on the following keywords: molecular dynamics simulations, visualization techniques, complex systems, proteins, review, survey. For our literature searches, we used the GoogleScholar search engine, the IEEEXplorer database, as well as journals including the Journal of Molecular Biology, the Journal of Molecular Graphics, Bioinformatics, the Journal of Chemical Information and Modeling, the Journal of Chemical Theory and Computation, the Journal of Molecular Modeling. For this, we combined different keywords with Boolean operators (AND, OR, NOT) to refine and narrow down our search results.

In the next step, we sorted the collected search results. When sorting, we focused on the articles dealing with the visualization of molecular complex systems and/or the visualization of molecular dynamics simulations. Then, we checked the articles that were cited in the originally selected articles. We used GoogleScholar to search for the relevant articles citing our first selection of articles. This brought us to a second level of sorting. All searches were combined, and we selected the articles that came closest to the defined scope.

To give the reader some orientation and structure, we have adopted a categorization that follows a first level with four axes of abstraction, which will be presented in a later section, describing temporal, scale, image and molecular dimensions. We have extended this categorization with a less formal second level of sub-structuring according to the emerging thematic groupings in the article corpus discussed in each section.

We have also examined previous surveys in this area, their structures and their perspectives. We found that none of them deal specifically with the visualization of the molecular dynamics of complex systems, which was another reason for compiling this review.

## 3 Brief history and related works

Molecular simulations have become a powerful tool for the study of biological phenomena. The last decade has seen impressive progress in large-scale molecular dynamics simulations. These simulations are usually based on experimentally determined initial structures. Advances in microscopy techniques, for example, have made it possible to push the experimentally available atomic-resolution descriptions of biological systems into the 1 million (10 nm) to 1 billion (100 nm) range. In fact, many works have presented simulations of complete ribosomes over increasing time scales (Brandman et al., 2012; Bock et al., 2013; Whitford et al., 2013; Sothiselvam et al., 2014). In 2019, (Singharoy et al., 2019), present a model at a 100-million atom scale depicting an entire cell organelle, specifically a photosynthetic chromatophore vesicle from a purple bacterium. Recently, (Casalino et al., 2021), investigated the infectivity processes of the SARS-CoV-2 spike protein with the entire SARS-CoV-2 viral envelope simulation, which includes 305 million atoms including 24 spike proteins with a simulation rate of 68 nanoseconds per day. In the same year, (Ozvoldik et al., 2021), Constructed all-atom models for SARS-CoV-2, HIV, and an entire presynaptic bouton with a diameter of 1 μ m and 3.6 billion atoms. They employed modular building blocks to markedly decrease GPU memory needs. In 2023, the scale of simulating an entire JCVI-syn3A minimal cell in full complexity was reached, modeled based on an integrative approach (Stevens et al., 2023). Whole-cell simulation is a major visualization challenge due to the complexity and size of the underlying molecular system. We are moving toward increasingly complex systems or giant molecular machines with an increasing number of simulation runs, over an increasingly long duration, each. We have reached a point where one can build larger, biologically underpinned molecular models than can be simulated. This is due, for example, to the memory and precision limitations of current codes, which are expected to be overcome soon.

On the other hand, only visualization of such MD trajectories, combined with other analytical tools, enables an immediate and intuitive comprehension of dynamics and function within these systems. However, a major part of molecular visualization techniques was designed for depicting static scenes. In the 1990s, both molecular visualization software and molecular dynamics simulation programs were developed. The common representations were adapted for the frame-by-frame visualization of trajectories. In the early 2000s, GPU acceleration, one of the most significant recent advances in visualization capabilities, was integrated into molecular dynamics visualization programs. This has made it possible to visualize molecular dynamics simulations with static frame-by-frame representations in real time, see, for example, the review of existing approaches, including among others those for static scenes, presented by (Kozlikova et al., 2017). Another review, presented by (Schatz et al., 2019a), discusses the progress of molecular visualization over the last decade, contextualizing the community’s advancements with the author’s contributions.

Aesthetics is another aspect of the visualisation of biomolecular structures that has progressed over the years in line with evolving technology, user requirements and methods of dissemination. (Mierzwa and Goodsell, 2021). employ two artistic methodologies to enhance scientific communication. The first involves the use of intuitive metaphors, facilitating novel connections and rendering complex scientific topics more accessible. The second embraces an integrative approach that consolidates diverse data sources into a cohesive visual representation. (Garrison et al., 2023). delve into the objectives, issues, and solutions that have influenced the contemporary state of biomolecular imaging, considering the viewpoints of informatics, structural biology, and biomedical illustration. They pinpoint both prospects and hurdles in shaping the future visual appeal of biomolecular graphics.

In the 2010s, virtual reality (VR) visualization was occasionally explored in relation to analytics in bioinformatics (Sommer et al., 2018) and molecular dynamics visualization programs. This technology allowed molecular dynamics simulations to be experienced in fully immersive VR environments, providing a more intuitive and interactive way to visualize such simulations. The application of immersive environments for the exploration of molecular structures, including trajectories, has recently been reviewed (Kuťák et al., 2023).

In the 2020s, the development of web-based molecular visualization tools has made it easier to access and use molecular visualization software from anywhere in the world (Sehnal et al., 2020; Sehnal et al., 2021). This expanded accessibility has opened new opportunities for collaboration and sharing of molecular dynamics simulations and visualizations (Pacheco et al., 2019; Kampfrath et al., 2022), but also brings its own technical challenges. The widespread use of notebook-like environments that rely on scripting languages to enrich raw simulation data has led to the integration of interactive molecular visualization widgets that facilitate data sharing and analysis. Pushing these approaches to scale up remains a current challenge.

The Deep Learning technique is very powerful to embed the complex high-dimensional molecular simulation data into a latent space with lower dimension that retains the inherent molecular characteristics (Doerr et al., 2017). To visualize such data, (Chae et al., 2019), presented an interactive visual analysis system for embeddings of MD simulations, aiming to assess and elucidate an embedding model while also analyzing various characteristics of the simulations. Recently, the integration of deep learning algorithms into molecular dynamics visualization programs has been observed, enabling the rapid emulation of photorealistic visualization styles from simpler, more accessible molecular representations (Durrant, 2022). This development has made it easier and faster to create animations that represent molecular dynamics simulations. While this approach does not allow for the precise representation of molecular systems that can be achieved with desktop tools for molecular visualization and analysis, it does allow for a significant acceleration of the process and produces results that are well suited for some scientific publications, outreach efforts and educational purposes.

**FIGURE 1:**
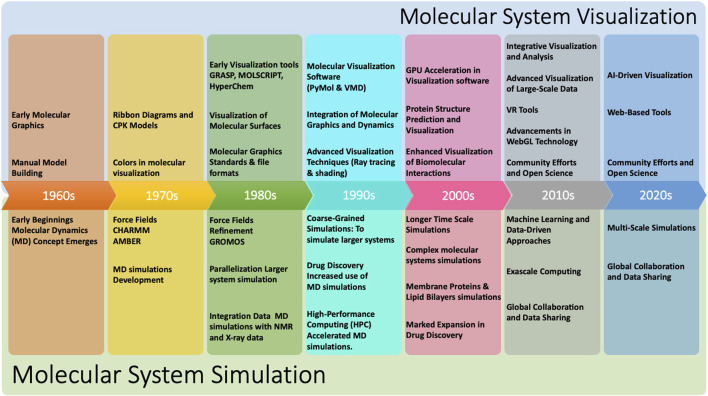
Timeline of selected historical milestones that have marked the development of the field of molecular systems visualisation and simulation (LEvTT et al., 1987; Van Gunsteren and Berendsen, 1987; Dyall et al., 1989; Humphrey et al., 1996; DeLano et al., 2002; Laxmi and Priyadarshy, 2002; Perilla et al., 2015; Olson, 2018; Case et al., 2023).

Figure 1 shows a continuous timeline of some of the milestones that have shaped the history of molecular systems visualization and simulation. Over the years, the power and functionality of molecular systems visualization programs have greatly improved, making it possible to visualize and analyze increasingly complex molecular systems such as whole cells and membranes. Furthermore, since it is rarely practical or useful to visualize all the atoms in such systems, rendering performance for the reduced representations that are of interest does not appear to be a major bottleneck at present.

In this report, we aim to study the visual representation approaches and metaphors that can be used to visualize large scale molecular dynamics simulations, taking advantage of the graphic and technological developments briefly mentioned in this section. A recent perspective article discussed similar aspects in the particular context of membrane systems, and we refer the interested reader to this work (Corey et al., 2023). In our review, we will prioritize areas not discussed in the more specialized perspective provided by Corey *et al.*

## 4 General considerations on visualization of complex molecular systems and their dynamics

The study of molecular dynamics simulations is very important to understand the behavior of molecular systems and identify all events of interest. Visualization critically assists scientists in identifying such events, particularly when they correspond to previously unknown patterns in the data that cannot be detected programmatically until they have been first characterized. In particular, visualization mechanisms that account for the temporal emergence of features are needed to provide fundamental insights into the dynamic behavior, transitions, and functional properties of these systems. Moreover, visualizing this temporal information will make it easier to spot interesting events. Frame-by-frame visualization using the classical representations and their combination has been the most common approach for decades. Indeed, biologists are very skilled at understanding the static structure of molecules using classical representation models and their combinations. They can therefore easily perceive and understand many aspects of dynamic behavior by observing the animated motions of these models.

However, with the latest data sets and in the wake of the exa-scale, direct replaying through the trajectory-data timesteps is no longer sufficient or workable. Indeed, molecular dynamics simulations now contain millions to billions of time steps of complex molecular machinery. In this section, we are interested in approaches that go beyond the standard stepwise rendering and could be used to visualize multiple aspects of such systems. In addition, this section describes efforts that can be utilized at the level of individual simulation data. In contrast, section 5 follows with a discussion of approaches that address visualisation of simulation ensembles and data sharing.

Finally, in order to guide the discussion, we propose a classification of these efforts based on the Visual Abstraction Formalism. What follows in this section is the description of this formalism in the context of molecular dynamics simulations, and four subsections corresponding to each of the axes of abstraction in our interpretation of the formalism: scale, temporal, molecular and image.

### 4.1 Visual abstraction formalism

Visual abstraction is a prominent key to visualize complex molecular systems in the context of dynamic simulations. It is a transformation that preserves the underlying concept in data visualization, converting information into visual depictions by eliminating details associated with natural variation, noise, etc. (Viola and Isenberg, 2017; Viola et al., 2020).

Initially, (Rautek et al., 2008), classified visual abstractions into “low-level” and “high-level” visual abstractions. Low-level abstractions deal with the representation of shapes and their appearance. High-level visual abstractions determine what to show and reveal the dominance of a particular structure in the resulting visualization. A strict separation, however, may be inadequate, as the work of van der (van Der Zwan et al., 2011) indicates the possibility of a seamless transition between low-level and high-level visual abstractions.

Later on, Viola and Isenberg (Viola and Isenberg, 2017) explored the application of the abstraction concept in scientific visualization. They discussed the different aspects of visual abstraction and considered the temporal aspects of a dynamic dataset conveying a given process or structural emergence. They proposed a structuring of visual abstraction that associates four axes of abstraction, namely object space, image space, temporal domain, and scale, with different spaces of integration.

**FIGURE 2:**
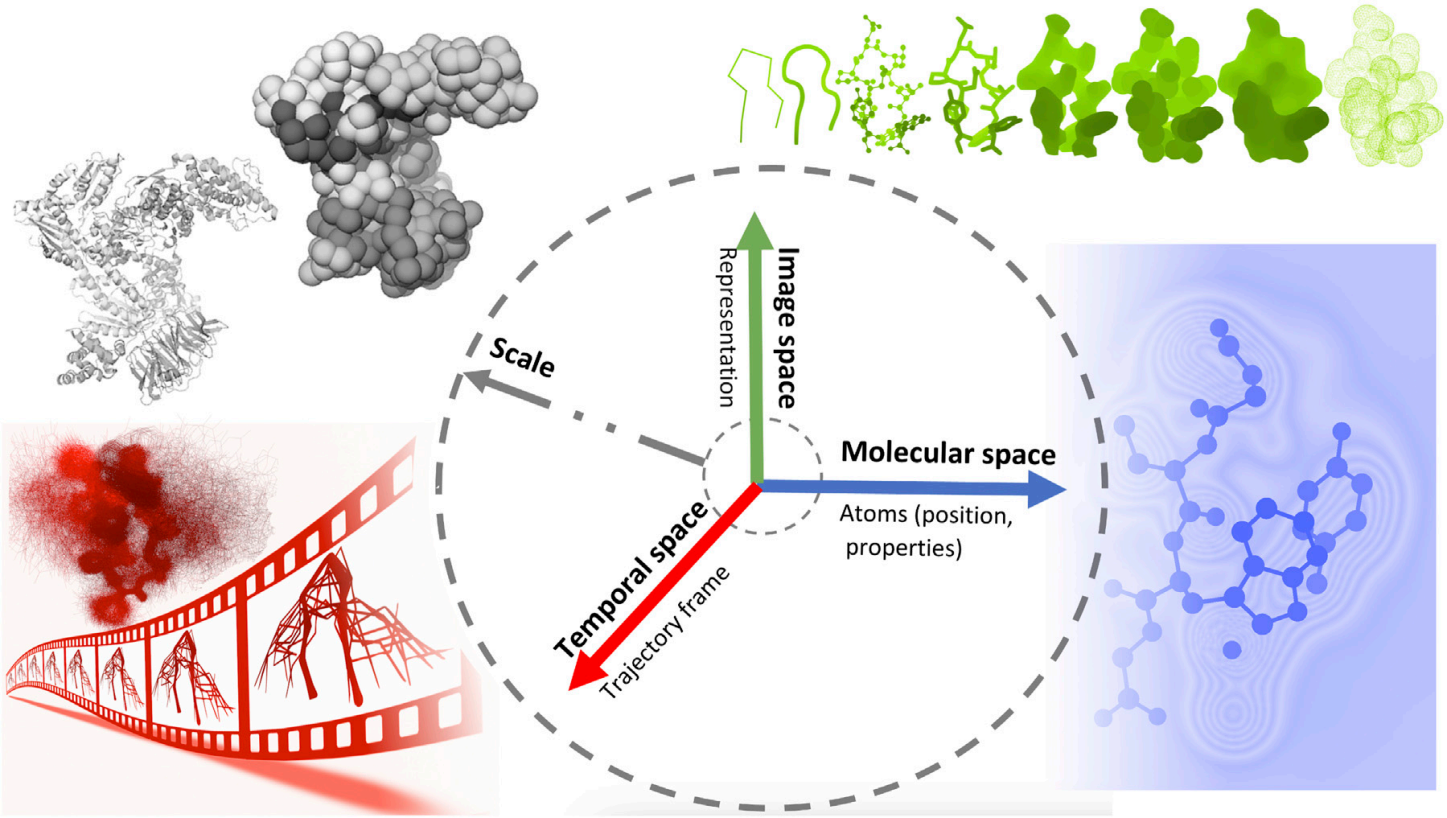
An illustrative representation of the visual abstraction space defined by four axes (scale, temporal, molecular and image axes), each associated with a specific space.

In this work, we propose a classification inspired by the structuring of Viola and Isenberg and adapted to the context of visualization of molecular dynamics. Figure 2 shows an illustrative representation of this visual abstraction space defined by four axes. The molecular abstraction space (object space) is associated with the molecular structure and properties, whereas the image abstraction space is associated with the representation of the structure. Finally, the temporal space concerns abstractions related to MD trajectories, whereas, the scale axis is linked to various levels of molecular details across these three axes.

To understand the relationship between adjacent scales, a visualization should convey properties that characterize each scale. A seamless visual transition in abstraction between these scales can be an effective way to convey their relationship to each other, which is a form of structural abstraction. Therefore, we present this classification with the awareness that each approach discussed here has an inherent overlap between the defined axes. At the same time, we hope that this somewhat arbitrary classification will facilitate the reader’s entry into the discussion and perhaps serve as a conversation starter that can be improved or refined.

### 4.2 The scale axis: multiscale visualization approaches

**FIGURE 3:**
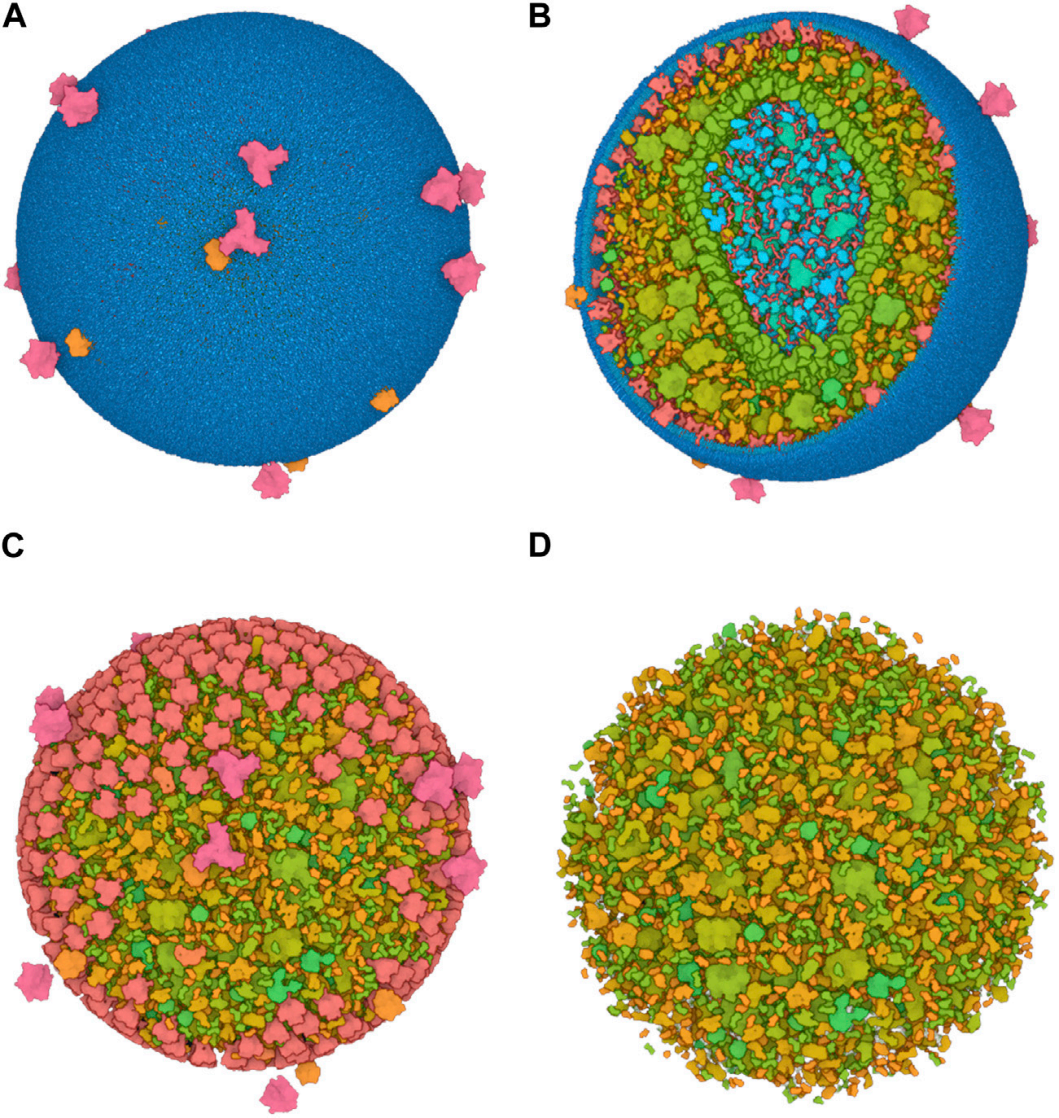
Example of multiscale visualization of HIV based on a combination of several levels of abstract representations and visualization frames: **(A)** Shows the overall abstract representation, where only the outermost scale is visible, and color highlights the inserted proteins that are easily identifiable compared to the membrane envelope; **(B)** shows a cut representation revealing the different scales that are efficiently separated by a combination of colors and shapes in a cross-sectional view; **(C)** is without the external surface representation, revealing what lies directly beneath the membrane or traverses it, with a marked color separation from the apparent cytosol just beneath; **(D)** is one scale of abstraction lower than **(C)**, revealing the cytosol. Images generated by CellView (Le Muzic et al., 2014).

To efficiently handle the increasing complexity of simulated large molecular architectures, specialized multiscale visualization approaches are very helpful (Miao et al., 2019). They are an efficient strategy to face up with complexity in various applications of molecular visualization. Currently, these are not usually included in tools for visualizing molecular trajectories, but are mostly found in visual exploration or model building steps. Typically, modeling biological phenomena spans various scales, encompassing individual atoms, proteins, and entire cells based on a combination of abstract representations. Scale and scalability are often used in scientific visualization to simplify the visual understanding of these systems. Figure 3 shows an example of a multiscale visualization of HIV, from the external envelope to the interior, with specific representations for each scale, generated by CellView (Le Muzic et al., 2014).

The multiscale models of complex biological assemblies contain macromolecular components of proteins, lipid membranes, and fibers of sugar polymers or nucleic acid strands. Dynamic models will be of particular interest in the future, but currently, static multi-scale models of these complex systems are available. Building such representations requires software tools created to construct models on a large scale. CellPACK (Johnson et al., 2014) for instance combines data from structural biology and systems biology with packing algorithms to construct detailed 3D models of cell-scale structures at the molecular level. To help illustrate these types of systems, CellPAINT (Gardner et al., 2021) enables users to generate depictions of the molecular structure of cells and viruses. It produces customized elements based on structure information from the Protein Data Bank (PDB) (Burley et al., 2022) and provides functions for interaction, grouping, and locking to craft illustrations of assemblies and complex scenes.

Achieving a good understanding of these systems requires a visualization strategy that considers the scale to be highlighted for a specific viewpoint and the suitable interaction metaphors at that level. If, for instance, structural details of a particular scale are accentuated without adjusting the viewing angle, the projection or zoom parameters might yield views that are excessively close or too distant. Consequently, the resulting image may either be overwhelmed with details that are challenging to comprehend or, on the contrary, may lack sufficient visual information.

**FIGURE 4:**
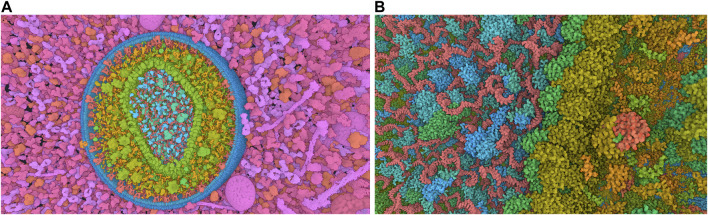
Virtual navigation at different scales of a HIV model in a blood plasma environment with a complete communication of structural details on each scale. **(A)** A cut plane representation of the HIV particle in the surrounding blood plasma. **(B)** Scale change via camera zoom from the same image viewpoint. The coloring adapts according to camera zoom to distinguish structures at actual scale. Images generated by CellView (Le Muzic et al., 2014).

The standard visualization modes for a given visual task include interactive 3D visualization of structures, allowing the user to freely explore the 3D scene while ensuring comprehensive communication of relevant structural details across all spatial scales of organization (Figure 4). Tasks related to managing visibility are used to overcome the occlusion of internal structures. This problem is determined by the inherent characteristics of the environment and the visual tasks involved such as object discovery and object access. (Elmqvist and Tsigas, 2008). presented a classification system for managing such 3D occlusion issues.

**FIGURE 5:**
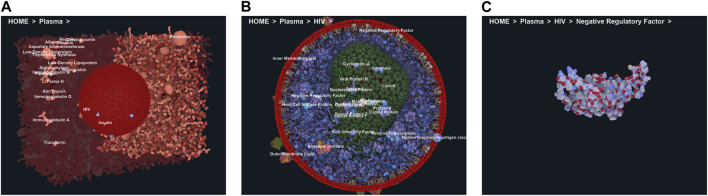
HyperLabeling of a Multi-scale HIV model in blood plasma. **(A)** Selection of the plasma HyperLabel to see the structure of the HIV; **(B)** Exploring the hierarchic HIV model through further HyperLabel interactions, **(C)** Last level shows a single instance of a viral protein. Image by under a CC-BY DEED 4.0 licence, reproduced from (Kouřil et al., 2020).

In a multi-scale visualization system, visual guidance is required. Coloring can be used depending on the scale at which the model is observed. (Waldin et al., 2016; Waldin et al., 2019). introduced a dynamic assignment of colors for multiscale systems, where color attribution depends on the hierarchical level and the count of visible structures in the actual view. (Kouřil et al., 2019). proposed a dynamic labeling method for intricate multiscale 3D molecular environments, wherein the scene hierarchy aligns with an associated label hierarchy. The label level for each region of the image is determined by the currently specified level of detail. Only the labels of visible structures are presented. A labeling system that gives more information about the selected element is implemented in the CellView program (Le Muzic et al., 2014). HyperLabels (Kouřil et al., 2020) is an approach designed for navigating hierarchical 3D models. It activates labels that, aside from their annotation function, also facilitate user interaction. Indeed, when a user clicks on HyperLabels, he chooses the following structure for inspection. Subsequently, the visualization adjusts to reveal the internal composition of the chosen subpart, allowing for continued exploration (refer to Figure 5). In 2021, Nanotilus, a guided-tour generator for exploring the biological nanoworld in an immersive 3D environment was introduced by (Alharbi et al., 2021). It transforms a 3D model and script (i.e., textual story), into an engaging guided tour, introducing a camera path planning technique and sparsification method to enhance user experience. In 2017, (Miao et al., 2017), described DNA nanostructure visualization across multiple abstractions with smooth transitions between levels of abstraction to provide continuous visualisation across scales.

Goodsell worked on integrative illustrations for the coronavirus, the minimal cell JCVI-syn3.0 and the microdomain of *Caulobacter crescentus* (Goodsell et al., 2020; Goodsell, 2022; Goodsell and Lasker, 2023). The main idea is to simplify visual complexity by employing streamlined representations, utilizing orthographic projection to preserve scaling relationships, and employing a color scheme that emphasizes the functional compartments of the molecular system. These works are geared towards accurately conveying the scientific achievements and ensuring usability in traditional media.

(Halladjian et al., 2021) introduce “Multiscale Unfolding,” an interactive approach to the visual depiction of several hierarchic scales of DNA in a single view. This technique allows viewers to interact with any scale at any time. The depiction is divided into “constant-scale zones” that preserve a “single-scale representation” and “scale transition zones” connecting adjacent zones through unfolding, scaling, and transparency. This approach enables for the spatial depiction of the complete DNA molecule, preserves local organizational features, linearizes higher-level organization, and offers coherent interpolation across different scales. The technique supports coarse-to-fine navigation and visual aids for illustrating size differences, allowing viewers to understand the structural composition of DNA from chromosomes to atoms in a single view. Still in the DNA context, Vivern (Kut’ák et al., 2021) is a virtual reality (VR) application designed for domain specialist to create and visually inspect DNA origami nanostructures. It offers abstracted visual representations, various color schemes, and the capability to examine multiple structures in a single environment, facilitating detailed analysis. The “Magic Scale Lens” and “DNA Untwister” tools enable experts to preserve context by embedding representations into local regions.

### 4.3 The temporal axis: aggregation approaches

Aggregation approaches involve the collection, standarization, integration, and analysis of data into a single element to reduce dimensionality. For example, spatial aggregation can be used to combine a group of atoms into a visual element or graphical representation that summarizes the information (see subsection 4.5). Beyond spatial aggregation, the inclusion of temporal aggregation exposes interesting features within the simulations that remain unnoticed when examining individual time steps. This form of aggregation empowers the user to comprehend the intricate dynamic behavior of selected components at the atomic level.

The simple superimposition of graphical representations from different time steps, sometimes called a cumulative view, is probably one of the oldest technique of temporal aggregation, alongside the works of (Rheingans and Joshi, 1999; Schmidt-Ehrenberg et al., 2002). Another classic example of spatio-temporal aggregation is provided by VMD’s volmap tool, introduced in 2006 (Cohen et al., 2006). In (Durrieu et al., 2008), the volume rendering was used to illustrate the water occupancy around a protein, averaged over a MD simulation. In 2011, (Thomaß et al., 2011), employed temporal aggregation of atom densities and their attributes to illustrate the average probability of the presence of mixed solvent components around a hydrogel. The visualization was executed using a color map on the average molecular surface of the hydrogel. At the same time, (Raunest and Kandt, 2011), developed dxTuber, a cavity detection tool that considers protein dynamics. MD simulations are employed to search protein cavities for solvent molecules. These cavities are characterised as domains with a high probability of solvent occupancy. In addition, each cavity’s total volume and cross-sectional area profiles can be computed.

In 2012, (Lindow et al., 2012), represented time-dependent channels of proteins using aggregation. For each coordinate set of the trajectory, the cavity structure is determined using the Voronoi diagram of the van der Waals (vdW) spheres. Then, the analysis of the time evolution of components of the cavity structure allows to calculate dynamic channels. For detailed exploration, a timeline visualization tool allows the user to select specific components of the cavity structures.

In 2014, (Chavent et al., 2014), developed a tool to visualize interactions between lipids and membrane proteins. They aggregated, on a grid, the diffusion movement of the lipids and used arrows and streamlines to visualize lipid dynamics. Later, they extended this work to filter and highlight dynamic and complex paths in molecular simulations (Alharbi et al., 2016). In 2020, (Pálenik et al., 2020), proposed an approach based on the partial aggregation of domains to facilitate the visual exploration of multiple scales. It simultaneously allows the construction of a continuous scale space for discrete data sets and the exploration of scales in space and time. The approach joins two scale spaces in a space-time cube and models the joined views as orthogonal slices through this cube. This approach enables quick identification of multi-scale spatio-temporal patterns.

In 2019, (Briones et al., 2019), introduced a computing package called GROma*ρ*s, for getting and analyzing time averaged density maps from MD simulations. Further toolsets can offer a multitude of analysis functions such as the computation of hydrogen bonding, root mean square displacement (RMSD), radial distribution function (RDF), contacts, interactions, topological descriptors and of course the density distribution as in (Allen et al., 2009; Gapsys et al., 2013; Giorgino, 2014; McGibbon et al., 2015; Esque et al., 2018; Gautier et al., 2018; Byška et al., 2019) presented an interactive method for visually analyzing extensive and complex molecular dynamics simulations. Their approach integrates a dynamic 3D focus + context visualization with a 2D time series chart, aiming to simplify the detection and examination of noteworthy spatio-temporal occurrences. The 3D visualization provides a more detailed representation of elements of interest, enhancing temporal resolution based on the time series data or the spatial region under consideration.

In 2022, (Skånberg et al., 2021), proposed an aggregation of the spatial distribution functions (SDF) in both temporal and molecular structure spaces from an ensemble of simulations. To achieve this goal, they developed an algorithm, based on Kabsch’s work (Kabsch, 1988), for tracking the internal frame of reference (IFR) that marked sets of points representing selected molecular structures.

(Eschner et al., 2023) explore the effectiveness of 3D animations in conveying complex biological processes. Their work focuses on the impact of smoothing motion in animations to draw attention to key elements, which can be seen as a particular form of temporal aggregation. A study with 108 participants compared geometric smoothing (reducing noise in motion trajectories) and visual smoothing (motion blur). The results indicate that moderate motion blur enhances the user’s capacity to follow the story, whereas geometric smoothing improves visual attractiveness. Both animation techniques, however, reduce perceived speed of the animation.

The techniques described up to now were generally applicable to a board range of molecular systems. A range of visualizations have been designed with specific cases in mind. Molecular cavities and pockets are of particular interest, as they govern transport phenomena, drug binding, ion permeation, etc. Several tools towards their trajectory-specific analysis and visualization have been developed and will be discussed in the next section.

### 4.4 The molecular axis: molecule-class dependent visualization

Molecule-class dependent visualization is a form of abstraction used for visually exploring long MD simulations on different scales (molecular space, see Figure 2). This visualisation focuses on providing information about molecule-specific characteristics, such as 1) molecular shape and inner cavities (e.g. pockets, channels); 2) molecular properties (e.g. charge, polarity); and 3) molecular interactions (e.g. chemical bonds, two-body and non-covalent interactions). All these characteristics are routinely studied and computed based on simulation data, and its visualisation is paramount to understand many relevant scientific questions. For example, the characterization of molecular cavities and protein-ligand interactions are used to assess the viability of new drugs during the discovery campaigns.

In order to facilitate the discussion and help the reader, this section is organized based on the aforementioned characteristics. First, we present work that predominantly focuses on the task of characterizing molecular shapes and cavities. Next, we discuss approaches that visualise molecular properties and finally, we mention efforts that address the study of molecular interactions.

#### 4.4.1 Visualisation of molecular shapes and inner cavities

**FIGURE 6:**
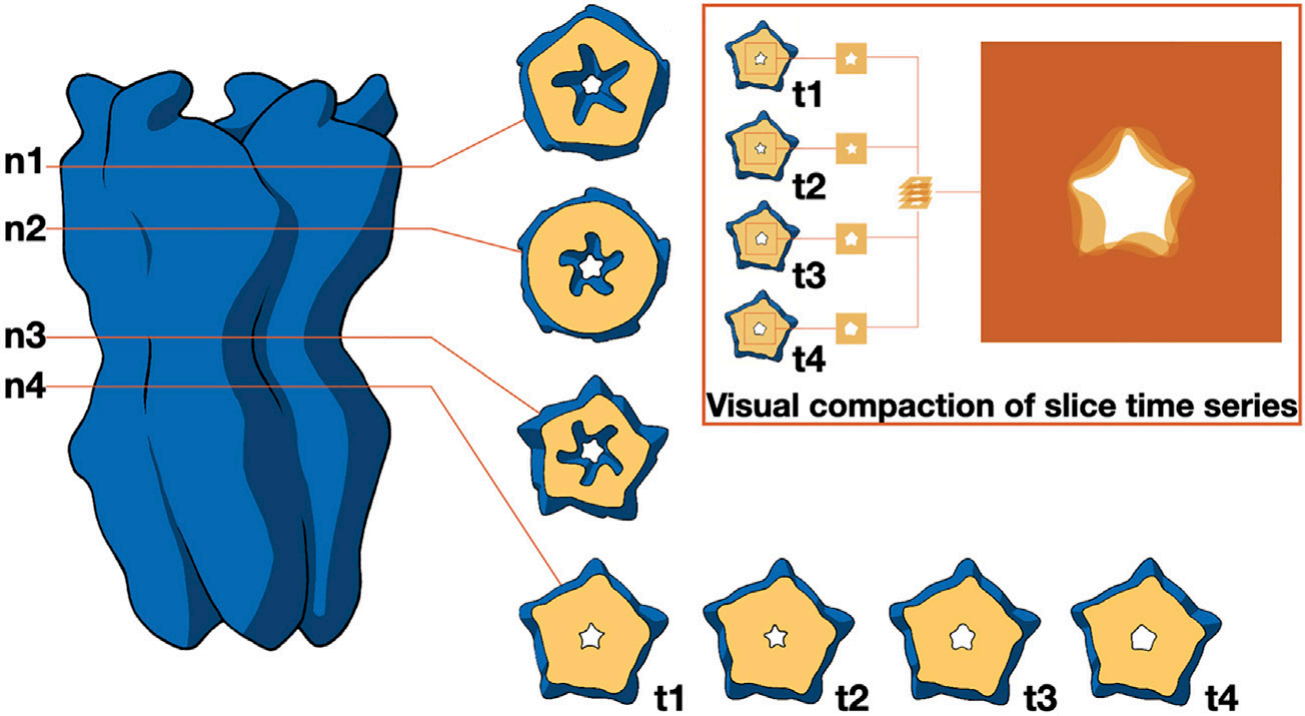
Distribution of ionic passages over time. It illustrates how the contour of permeation pores for ions can be identified through cross-sections at varying heights (n1 to n4) relative to the membrane. The primary constriction at n4 is observed across a series of time points (t1 to t4), which is visually summarized by overlaying transparent images. Image by Spalvieri et al., 2022, under a CC-BY DEED 4.0 licence, reproduced from.

As mentioned before, the study of molecular shapes is typically intertwined with that of molecular interactions (see below). However, it is worth highlighting the more general work of (Spalvieri et al., 2022), which discusses the utilization of design principles to experiment featuring abstract shapes with exploded views of molecular structures and motion-averaged slices. In their work, the authors investigate the ways in which design enhances the fields of structural biology and bioinformatics, demonstrating how designers contribute to making scientific data accessible without distorting its meaning. In the context of motion-averaged slices, the authors employed a lateral contour to identify significant channel constrictions, serving as reference points to generate a contour slice, as depicted in Figure 6. The pivotal constriction (n4) was tracked throughout a molecular dynamics simulation via a time series of contours, which were visually condensed by overlaying individual images with suitable transparency levels. The resultant 2D image encapsulates the temporal progression.

As previously suggested, a lot of works have been devoted towards the visual exploration and understanding of the ligand and its environment. In most cases, ligands are small molecules, but one may also consider other macromolecules as “ligands,” for instance when thinking about complexes such as the nucleosome (Elbahnsi et al., 2018). On the other hand, the environment is usually another macromolecule (protein), which exhibits distinct geometrical features in the form of cavities, where the ligand can be located (pockets) or traverses through (channels).

(Byška et al., 2015) proposed prospecting tunnels, from the outer protein environment to the active site. This technique allows the visualization of the dynamics of a single tunnel. They employed a dedicated heat map to provide an overview of all identified tunnels in the dynamics, showcasing their bottleneck width and stability over time, or a particular tunnel in the dynamics, displaying the bottleneck position and variations in tunnel length over time. These techniques aid in the selection of a limited subset of tunnels, which can be thoroughly examined individually in detail.

Another tunnel exploration technique is the AnimoAminoMinner (Byška et al., 2016). It enables the depiction of a complex 3D structure and its curvature details through a straightened centerline of the tunnel and its associated width profile. Each amino acid is illustrated by an ensemble of colored lines that represents the spatial and temporal influence of the amino acid on the respective tunnel. This representation highlights the significance of amino acids based on specified criteria. (Furmanová et al., 2017a). proposed a multiscale simplification model of the ligand trajectory, thereby, the user can see the global ligand movement within the protein. More work on protein tunnels and ligand exploration has been carried out (Lindow et al., 2011; Kozlíková et al., 2014; Kolesár et al., 2016; Furmanová et al., 2017b).

In a separate approach, (Malzahn et al., 2017), introduced a reprojection technique that transforms the 3D structure of a molecule into a 2D representation of the protein tunnel interior. This transformation retains individual residues, allowing the resulting 2D representation to unveil binding properties within the tunnel without encountering occlusion issues.

(La Sala et al., 2017) propose an automatic approach that enables the detection of potential pockets and allosteric communication networks in long molecular dynamics simulations. Utilizing NanoShaper (Decherchi, 2012), the algorithm traces the evolution of identified pockets on the protein surface during an MD trajectory. They highlight that dynamic analysis of pocket dynamics improves mechanistic knowledge, suggesting its potential application in characterising “transient binding pockets for structure-based drug design.” (Decherchi et al., 2019). present the interfacing of NanoShaper within their VMD tool (Humphrey et al., 1996), highlighting NanoShaper’s ability to identify and visualize channels, expanding its range of applications.

Molecular Sombrero is an abstract visualization method for protein binding sites, proposed by (Schatz et al., 2019b). This representation depicts the cavity and the surrounding surface region with a hat-shaped form, where the brim symbolizes the surface, and the crown signifies the cavity. This depiction is less crowded compared to traditional molecular surface visualizations. The simplification emphasizes crucial factors, including the cavity diameter, and simplifies the process of comparing various datasets side by side.

In 2020, (Bedoucha et al., 2020), investigated protein function through an interactive visualization approach to reduce and explore the vast data space. This method enables the deduction of pertinent protein function by analyzing the dynamics of a single structure through protein tunneling analysis, taking into account combinations of normal modes that span the entire normal mode space. Similar to the aforementioned example, the authors utilize a variety of interconnected 2D and 3D views, facilitating swift and adaptable exploration of individual modes and their impact on the dynamics of tunnels relevant to protein function. Once a noteworthy motion is pinpointed, the investigation into potential normal mode combinations is guided by a visualization-based recommendation system. This permits the identification of a concise yet significant set of normal modes that can be thoroughly examined. In a study by (Guo et al., 2020), a series of scales and visualizations of cavities is constructed, incorporating temporal and spatial perspectives to empower domain experts to manage their work at any level of semantic abstraction. These scales illustrate the chemical and structural properties of cavities, ranging from an entire protein to a cavity at a specific time in both temporal and spatial dimensions.

#### 4.4.2 Visualization of molecular properties

Most visualization tools (see section 6) allow to highlight certain molecular properties, such as hydrophobicity, polarity, and electrostatics by colour encoding onto the graphical representations. However these are not designed to be used for trajectory visualization. As these properties are not continuous between frames, even if a frame by frame adaptation is possible, it may not visually make sense because of the discontinuities.

Many properties can be mapped onto molecular surfaces. One important bottleneck that was lifted with approaches such as QuickSurf based on Gaussian surfaces is the capacity to generate such surfaces in real-time (Krone et al., 2012). Similar approaches have been presented for the Solvent Excluded Surfaces (SES) (Hermosilla et al., 2017a; Martinez et al., 2019a; Schäfer et al., 2019; Lindow et al., 2010) proposed various enhancements, based on a parallelization approach, aimed at reducing update times for the SES and the molecular skin surface (MSS) visualization.

While current GPU-based rendering techniques offer high frame rates for SES visualization, rendering large molecules or complexes may surpass hardware memory limitations. (Rau et al., 2019), propose CPU ray tracing as an alternative, achieving interactive frame rates without strict memory constraints. Rendering extensive molecule complexes is achievable with just a prior individual precomputation of Solvent Excluded Surfaces (SES). The results suggest that adopting a straightforward instancing approach for geometry effectively minimizes memory consumption, allowing for the exploration of significantly larger molecular datasets. Such techniques could in principle be combined with most of those described in the following paragraph for efficient trajectory analysis.

Approaches for mapping information onto molecular or generated surfaces are very diverse. Cipriano and Gleicher (Cipriano and Gleicher, 2007) used surface marking to represent charges over the molecular surface and thus enhance comprehension. Falk et al., 2009 highlighted the molecules’ reactions by arrows and stored the position of the reactions during the simulation for a subsequent analysis. Vesta (Momma and Izumi, 2011) and Haschka (Haschka et al., 2015) represented electrostatic potential by colouring the isosurfaces of electron densities. (Dahl et al., 2012) proposed the visualization of heatmap graphics according to the local geometry of the alpha helix axes. Khazanov and Carlson (Khazanov and Carlson, 2013) represented the interaction between ligand and binding sites by color modification and van der Waals radii at an atomic scale. Consequently, augmented radii and warmer colours indicate more contacts. On a different note, (Scharnowski et al., 2014), proposed the utilization of deformable models to establish a mapping relation between two surfaces. This method combines the difference value and comparability, deduced from the local matching quality in a 3D molecular visualization, by translating them into color representations.

**FIGURE 7:**
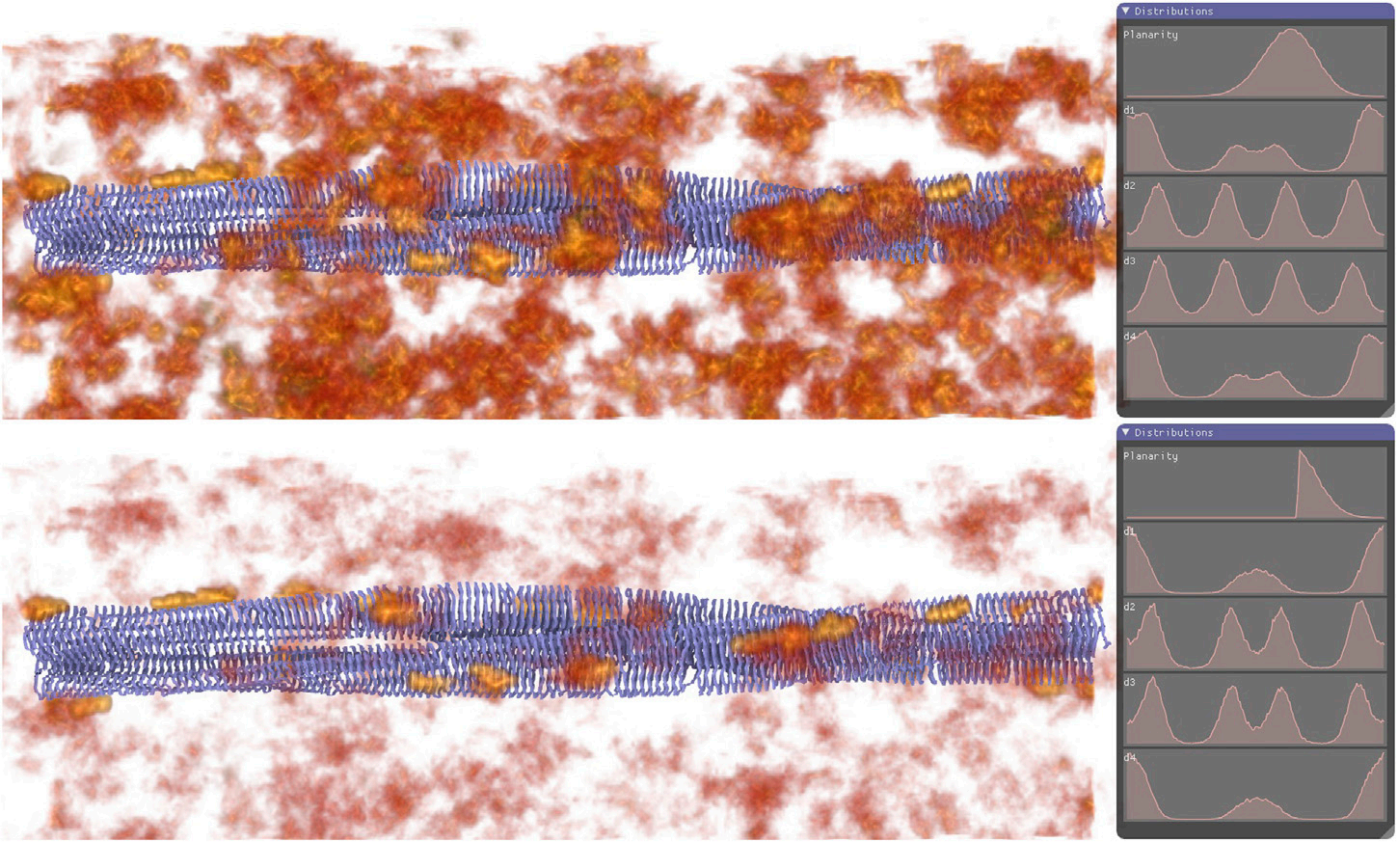
Illustrating the amyloid fibril through ribbon representation and depicting probe atom occurrences via the density volume (ranging from red to yellow). In the top figure, no filters have been applied, whereas the planarity range of interest has been identified from the distribution view and subsequently filtered in the bottom figure. The interesting spatial locations emerge upon observing the movement density field of the filtered occurrences. Image by under Creative Commons licence, reproduced from (Skånberg et al., 2018).

In 2018, (Skånberg et al., 2018), developed the VIA-MD environment for the visual interactive analysis of molecular dynamics, employing a semantic description that allows users to specify sets of molecular properties. VIA-MD seamlessly combines a spatial view for observing the geometric arrangement of molecules with a temporal time series view that displays events occurring over time. The authors presented four case studies, one of which centered on simulating amyloid plaque associated with Alzheimer’s disease development. Their focus was on amyloid fibrils comprised of misfolded beta-amyloid proteins, and their binding to these fibrils. In Figure 7, the amyloid fibril is depicted by ribbons to ensure visibility without obstruction, while still providing structural context to the density volume. The resultant density volume reveals a series of “hotspots” along the surface of the amyloid fibril, leading the authors to infer that p-FTAA molecules tend to exhibit greater planarity in proximity to the amyloid fibril.

Subsequently, Moliverse was introduced by (Brossier et al., 2023), integrating the VIA-MD molecular visualization framework into the OpenSpace astronomical visualization software (Bock et al., 2019). This integration facilitates the smooth connection of two markedly different scales, enabling the representation of phenomena such as gas composition in a planet’s atmosphere or molecular structures in comet trails.

Recently, (Ulbrich et al., 2023), presented sMolBoxes featuring a dataflow model designed for exploring and analyzing extended MD simulations. Through the integration of quantitative analysis for user-specified properties and interactive 3D visualizations, it facilitates the visual interpretation of molecular behaviors. Operating on a node-based model, sMolBoxes allows for the flexible definition, combination, and prompt evaluation of properties under investigation. Each sMolBox swiftly provides insights into observed properties or functions, contributing to an efficient discovery process.

#### 4.4.3 Visualisation of molecular interactions

Molecular interactions are typically quantified by a numerical value, or energy. As such, many visualization tools show interaction energies through 2D views that display energies with the corresponding (pair) interaction. For example, PoseView (Stierand et al., 2006), LigPlot + (Laskowski and Swindells, 2011), LeView (Caboche, 2013) and Maestro (Schrödinger, 2015) generate two-dimensional interaction diagrams of complex three dimensional structures. These tools rely on a close contact analysis with minimal interactivity, lacking the capability to filter molecular interactions based on their strength or distance.

In 2017, (Hermosilla et al., 2017b), introduced visualizations with the purpose of facilitating the characterization of interaction forces. They put forth a visualization concept tailored for analyzing binding forces in the fields of drug design and protein engineering. Employing a GPU-based suite of programs, the system calculates an array of energies for each residue. Various visualization motifs and filters are incorporated into the system, streamlining the process of data analysis. Similarly, (Vad et al., 2017), delved into the interactions between the protein and solvent molecules, employing highly abstracted 2D views to observe the overall behavior of solvent molecules.

(Furmanová et al., 2018) present innovative techniques for visually abstracting protein-protein interactions (PPI) configuration space, aiming to guide the exploration process. This visual representation assists experts in proteomics by facilitating the selection of pertinent configurations and the examination of contact zones at various levels of detail. The system incorporates methods that align with proteomics workflows. The Matrix view employs interactive heat maps to provide a comprehensive overview of residue-residue contacts in PPI configurations, allowing users to apply interactive filters. Subsequently, users can navigate through input configurations to understand interacting amino acids. Detailed information about amino acids within contact zones is presented in a Contact-Zone list-view, serving as a comparative tool for ranking models based on their similarity to template-structure contacts. The “Exploded view” and the “Open-Book view” represent individual configurations in three-dimensional space, resolving the challenge of high overlap associated with numerous configurations.

In their work, Vázquez et al., 2018 introduced a framework designed for the concise 2D representation of molecular simulations. The system encompasses a collection of 2D Information Visualization (InfoVis) tools that incorporate coordinated views, effective interaction capabilities, and focus + context techniques, all tailored for the examination of extensive datasets. Offering an array of patterns for entities like protein secondary structures or hydrogen bond networks. The system also includes interactive inspection tools, facilitating the exploration of a single simulation or the comparative analysis of two distinct simulations.

(Alharbi et al., 2019a) present a conceptual framework for visualizing interactions between proteins and lipids (PLI) as well as protein-protein interactions (PPI) in Molecular Dynamics simulations. The inherent challenge involves comprehending the dynamic nature of these interactions within simulations characterized by size, duration, and complexity. The framework they propose incorporates four interconnected perspectives: a time-dependent 3D visualization, an hybrid view, a clustering timeline, and a details-on-demand window. Diverse visual approaches, such as a hybrid 2D space, a projected tiled space utilizing a heat-map style for PLI and PPI at the particle level, and the use of glyphs for PPI at the molecular level, are implemented. This framework facilitates the study of PLI and PPI either independently or collectively through a unified visual analysis. Subsequent work focused on PLI visualization was conducted by (Alharbi et al., 2018; Alharbi et al., 2019b).

In 2019, (Duran et al., 2019), propose an interactive system designed for visually exploring exceedingly large trajectories, the data automatically generates various visualization patterns to provide users with insights into protein-ligand interactions. The system incorporates dedicated widgets to streamline and expedite data inspection and navigation to interesting segments of the simulation. It proves particularly effective for simulations featuring multiple ligands. Still in the context of interactive visualisation and the analysis of large trajectories, (Jurčík et al., 2019), propose to study a set of ligand trajectories encompassing various interconnected 2D and 3D views, facilitating the interactive exploration and filtering of trajectories with a knowledgeable approach. The authors elaborate on how drill-down techniques can be utilized to generate and save selections of trajectories with specific properties, allowing for the comparison of multiple datasets.

In that same year (Fassio et al., 2020), present a web server named nAPOLI (Analysis of PrOtein-Ligand Interactions). It enables atomic-level analysis of protein-ligand interactions and provides exhaustive reports on residue/atom interaction. For visualization purposes, nAPOLI supplies an interaction viewer that allows users to observe the complex and its interactions interactively, presenting 3D and 2D views of the complex. The tool includes a graphical analysis feature with statistical reports on the frequency and distribution of each type of atom and interaction. Additionally, (Furmanová et al., 2020), introduce DockVis as a visual analysis tool designed to work with multivariate output data from CaverDock (Filipovič et al., 2019). DockVis provides interconnected 2D and 3D views, allowing to comprehend the spatial configurations of ligands as they traverse protein tunnels. The tool guides users to pertinent segments of ligand trajectories, streamlining detailed exploration in subsequent analyses.

**FIGURE 8:**
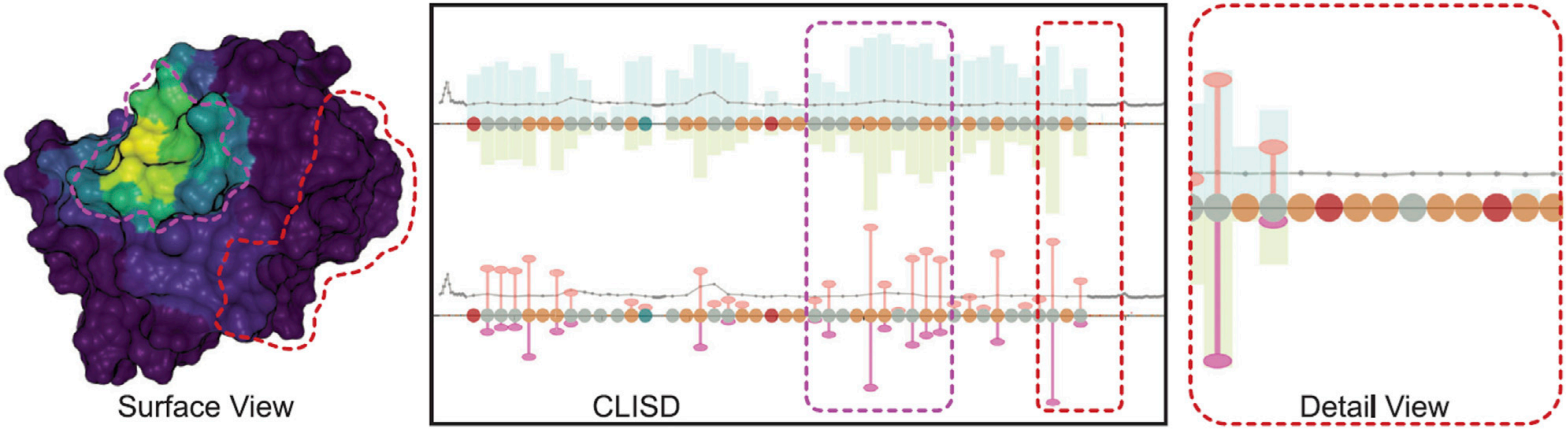
Visualization of protein-ligand interaction in three perspectives: the *x*-axes depict the amino acids constituting the protein. Above the *x*-axes, bars indicate the frequency of time steps in proximity to the ligand molecule, while below, they represent the number of near ligand atoms. The width of the bars is adjusted in proportion to the value, thereby highlighting higher, more significant values. The surface view is coloured with various aggregate quantities using the Viridis color map, where purple denotes zero and yellow indicates the highest value. Image by, under Creative Commons licence, reproduced from (Schatz et al., 2021).

Finally, in 2021 (Schatz et al., 2021) introduce an approach for studying protein-ligand interactions by focusing on the overall behavior of the system rather than individual ligand molecules. To address the challenge of dealing with simulations involving millions of time steps, the authors propose a system that separates visualization and data preprocessing. Ligand movement relative to a receptor protein is aggregated in the preprocessing pipeline. The web-based visualization application, integrates interconnected 2D and 3D views, featuring a sequence diagram presenting computed values linked to a conventional protein’s surface visualization (Figure 8). This interactive visual representation is crafted to remain unaffected by the scale of the underlying data, maintaining a consistently low memory footprint, even when dealing with input data in the order of several terabytes.

### 4.5 The image axis: visualization specifically for molecular dynamics

The image axis refers to the visual abstraction associated with the representation of the molecular structure. This visual abstraction has a multiple purpose, as it provides clarity by reducing the complexity of a scene, while also adding information. Most graphical representations and visualization techniques for molecular systems were not developed specifically for visualizing molecular dynamics. Nevertheless, we have been able to identify some approaches that have been designed and developed for the visualization of molecular dynamics.

Simple visualization of the snapshots representing the state of the molecule in some time steps is not sufficient because it does not provide a smooth animation. Using an interpolation between meshes representing two snapshots leads to an enormous number of triangles. Because of this increase in primitives, real-time animation of the movement is very hardware-dependent. In 2009, Zamborsky et al., 2009 propose a smooth animation of the Ribbon representation in real-time and enable the user to shift the animation slider in order to explore whatever part of the animation. They create the patterns of the beginning, middle and end part of each secondary structure. The pattern representing the middle part is then replicated as many times as necessary to create the secondary structure of the desired size. Then, the animation can be processed only by following the movements of the protein backbone (C*α* atoms), which notably simplifies the whole process of animation.

In 2011, (Wahle and Birmanns, 2011), developed a method to speed up ribbon and cartoon visualizations. This approach involves calculating a continuous curve along the protein’s main chain on the CPU. Subsequently, the GPU independently moves vertices along that curve to establish the final molecule’s geometric representation. In 2015, (Hermosilla et al., 2015), developed an algorithm that renders the geometric characteristics of the secondary structure of highly complex proteins using the tessellation stage of the GPU. The algorithm uses a B-Spline (Carson, 1991) to model the protein’s backbone and is based on patches to generate ribbons in real time. For every residue atom, two rectangular patches with opposing orientations depict each segment of the backbone. It can generate only the necessary geometry for the current viewpoint, facilitating real-time interaction with larger molecules.

**FIGURE 9:**
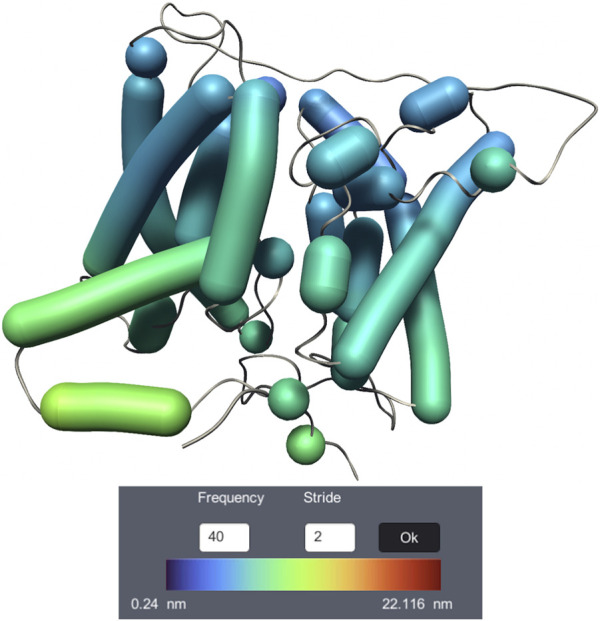
Visualization of membrane transport protein UT-B Urea dynamics, using the Dy-Bendix representation.

Dy-Bendix (Dynamic Bendix) (Belghit et al., 2023) adds a novel feature to the Bendix representation (Dahl et al., 2012), dedicated to molecular dynamics visualization of *α* helices. It is developed inside UnityMol (Lv et al., 2013). Dy-Bendix considers *α* helices’ fluctuation to quantify the movement amplitude of *α* helices during the molecular dynamics trajectory. This fluctuation is represented by a coloured heatmap on the Bendix surface. Even on a static image, the colour of the heatmap allows to detect large fluctuations in the helices. Figure 9 shows the membrane transport protein UT-B Urea with the Dy-Bendix representation. The helices with red colors indicate a big movement. The more the heatmap color tends toward blue, the less movement is observed. The color spectrum is displayed on the Dy-Bendix interface with the calculated threshold amplitude values.

**FIGURE 10:**
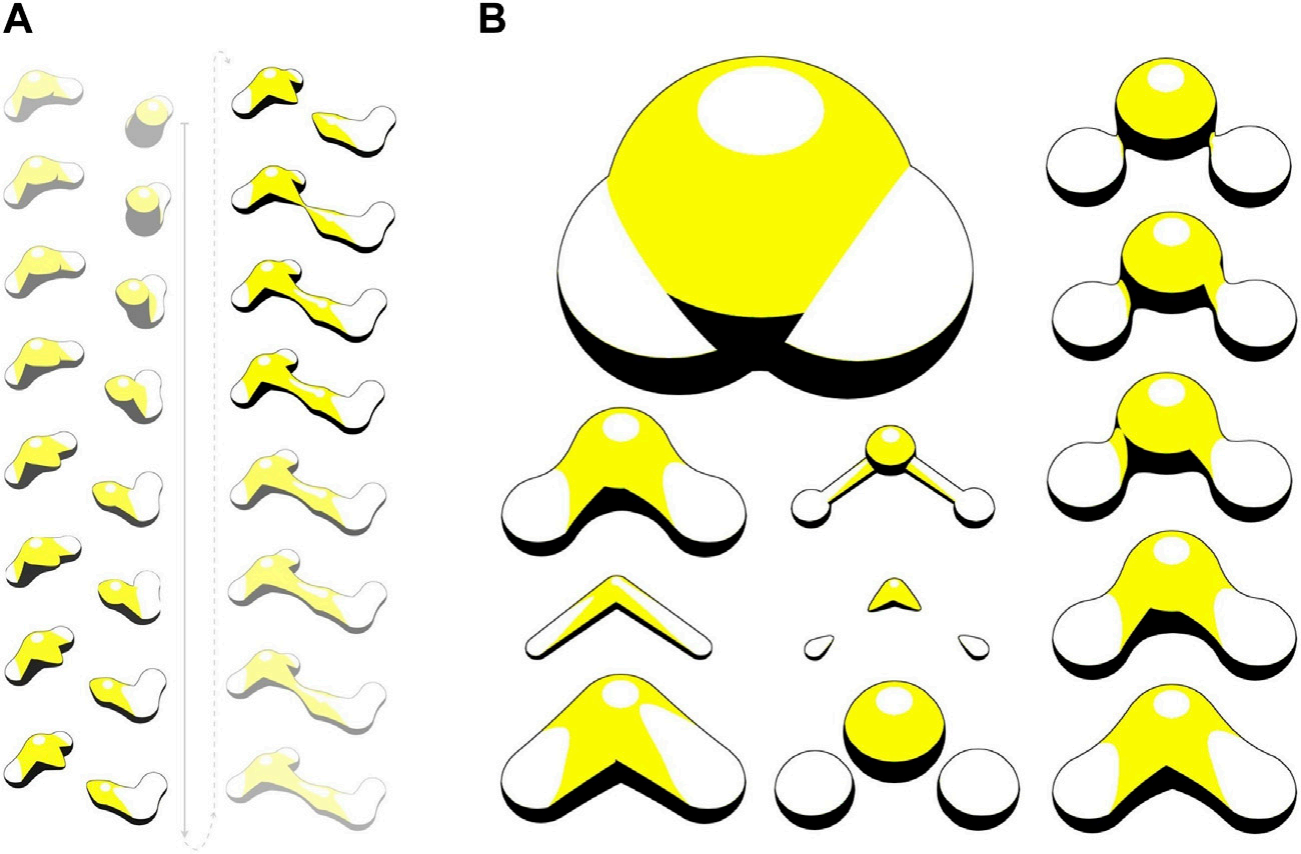
HyperBalls ray-casting to visualize molecular graphics in interactive simulations. **(A)** Visualisation of bonds between two water molecules. **(B)** Scale and shrink factor tuning. Image taken from (Baaden, 2023).

HyperBalls (Chavent et al., 2011) is one of the rare representations that have been designed to intrinsically take dynamics into account. HyperBalls introduces an enhanced ball-and-stick representation, substituting tubes with hyperboloids to smoothly connect the atom spheres. In Figure 10, the yellow structures illustrate bonds between two water molecules. HyperBalls indicates the bond direction through disconnected hyperboloid expansions of atoms involved. This form of representation is especially valuable for illustrating dynamic phenomena, like the progression of noncovalent bonds. Additionally, it is well-suited for depicting coarse-grained models and spring networks.

TRAPP (TRAnsient Pockets in Proteins) (Kokh et al., 2013) is an automated platform designed to track, analyze and visualize variations in binding pockets throughout a protein trajectory or within an ensemble of protein structures. These variations may encompass conformational changes ranging from local chain fluctuations to global backbone movement. In the same vein, POVME (Wagner et al., 2017) is an open-source binding pocket analysis software that is capable of comparing extensive pocket ensembles derived from MD simulations. It generates outputs conducive to quantitative analysis. Additionally, it facilitates pocket selection for ensemble docking and incorporates a chemical coloring scheme for binding pockets, allowing users to define pocket regions based on the ligand molecule’s position. When performing such analysis on large ensembles of molecular dynamics trajectories, performance can be an issue. The ePOCK (Laurent et al., 2015) tool was specifically written to optimize this process. It can produce simple visualizations of a pocket trajectory. More sophisticated analyses of complex dynamic pocketomes have been implemented in the mkgridXf tool (Monet et al., 2019).

(Bok et al., 2022) present a visual analysis sofware for MD simulations, utilizing dimensionality reduction to project conformational changes onto a 2D domain. A scatter plot reflects conformational differences, aiding in the identification of semi-stable conformations through clustering. The application allows users to compare 3D protein structures at different time points in a side-by-side view. Multiple simulation datasets were tested for validation.

SynopSet (Luo et al., 2022) is a set of visual abstractions for inspecting molecular dynamics simulations in DNA nanotechnology. It repurposes a progress bar and introduces new visuals. The authors create representations capable of displaying spatial and temporal details at various levels of abstraction. The set allows for smooth transitions between representations, facilitating efficient analysis at different temporal resolutions. The visualization space of the tool comprises three different axes (idiom, granularity, and information layout), which allows the systematic localization of the representations set. Similarly, (Lindow et al., 2019), present a ray casting-based visualization of RNA and DNA secondary and tertiary structures, which enables for the first time real-time visualization of even large molecular dynamics trajectories.

**FIGURE 11:**
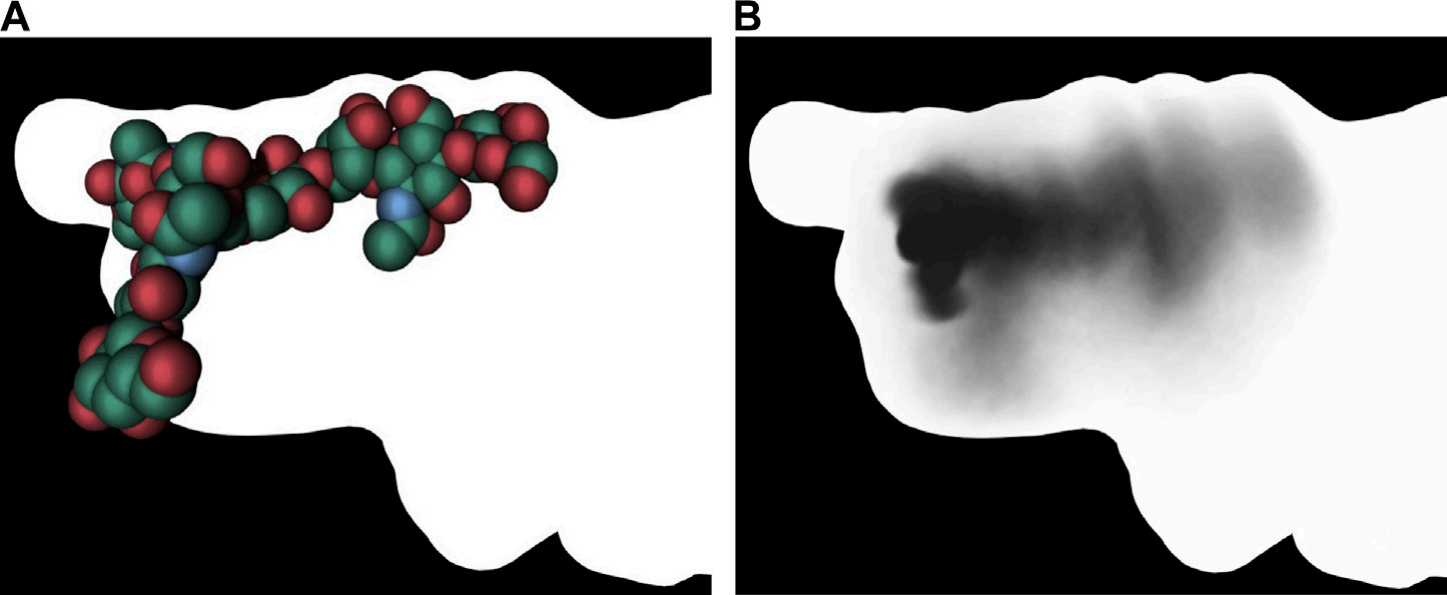
Umbrella visualization of the glycan. The left image shows the glycan at a single frame using vdW visualization mode. The right image shows the statistical shadow corresponding to the projection, on protein surface, of different glycan positions along the trajectory (Besançon et al., 2020a). In both images, the surface of the insuline receptor is rendered with lighting that removes any depth effect.

Coarse-grained (CG) representations nowadays represent a very common approach for reducing the cost of molecular dynamics simulations while preserving the physical/chemical sense of the fundamental interactions. However, the variety of CG representations, such as sizes, connectivity and naming, hinders the standardized handling and visualization of such models. Literature on coarse-graining approaches is abundant. We will here only skim over a handful of articles that give some thought to the visualization aspects and have not been discussed in (Corey et al., 2023), where coarse-graining is very frequent. SIRAH (Machado and Pantano, 2016; Klein et al., 2023) comprises a set of utilities that includes the conversion of all-atom coordinates to arbitrary residue-based CG schemes and features a VMD plugin designed for visualizing, analyzing, and extracting pseudo-atomistic details from coarse-grained trajectories conducted using the SIRAH force field. Recently, (Marchetto et al., 2020), proposed a web platform for preparing, running, and analysing coarse-grained molecular dynamics (CGMD) simulations. This platform is capable to show the simulation run directly within a web browser, using NGL Viewer (Rose et al., 2018). The UnityMol software has some coarse-grained specific features, such as visualization of secondary structure guides for proteins and nucleic acids, a functionality that most visualization tools currently lack for CG systems.

(Besançon et al., 2020a; Besançon et al., 2020b), developed an original approach in which a shadow captures certain features of a molecular simulation. This umbrella visualization mode is a graphical method developed in the context of sugar simulations. The approach assigns intrinsic flexibility to glycans during a molecular dynamics trajectory. The authors have integrated this analysis method into a visualization mode within UnityMol (Lv et al., 2013). The umbrella visualization considers both the primary positions of each glycan antenna and their statistical significance. By depicting the collected data as a shadow projected onto the protein surface, one can study protein/glycan interactions and gain insight into how glycans shield certain protein surface sites (Figure 11).

**FIGURE 12:**
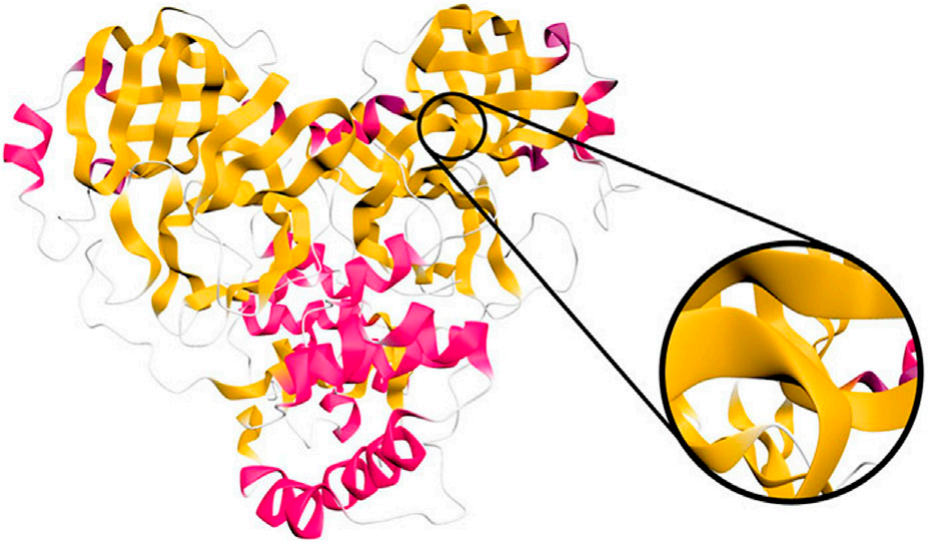
Narupa iMD rendring of the ribbon representation of the SARS-CoV-2 main protease (PDB ID: 6W63). Image by under a CC-BY DEED 4.0 licence, reproduced from (Jamieson-Binnie and Glowacki, 2021).

An interesting aspect to consider is that a human viewing a molecular trajectory may be distracted from interesting phenomena if visualization artefacts are present that may capture attention. In 2021, precisely in this context, (Jamieson-Binnie and Glowacki, 2021), worked on the visual continuity of the Ribbon diagrams when viewing a molecular trajectory. In order to avoid a distractful flipping and twisting, the authors suggest a novel method that avoids this artifact by morphing between consecutive cross sections instead of rotating (refer to Figure 12). This approach results in diagrams that are particularly suitable for observing dynamic simulations.

A last element concerns how to communicate results from molecular dynamics simulations visually. The primary medium of scientific communication remains the literature, with printed or online figures that are for the vast majority limited to 2D. How one can capture the intrinsic dynamics in such a limited medium remains an unsolved question that has been discussed elsewhere already (Kozlikova et al., 2017; Martinez et al., 2019b), but calls for more visualization research in that direction, and is particularly stringent for MD simulations. A better way than 2D images, that still needs to find its usage into current academic practices, is to share the actual visualization experiences more widely (Martinez and Baaden, 2021) and with various degrees of immersion. The issue with depicting dynamic aspects further alludes to the intrinsic noise and uncertainties in such simulations, that we do not know how to efficiently visualize yet, although some fundamental work is made in this direction (Schulz et al., 2018).

We have found that the major advances in molecular dynamics visualization are related to the analysis aspect and technological and computer science developments, such as the integration of GPU support and virtual reality into many software and web visualization platforms. However, with respect to graphical representations, there is very little work that specifically addresses MD visualization. We believe that the next-generation of visual representations must be inspired by the dynamics of molecules. An essential aspect for such approaches to be available to the community is their translation into viable software tools. For more details, section 6 discusses common tools and software in this area.

## 5 Scaling up to simulation ensembles and data sharing

**FIGURE 13:**
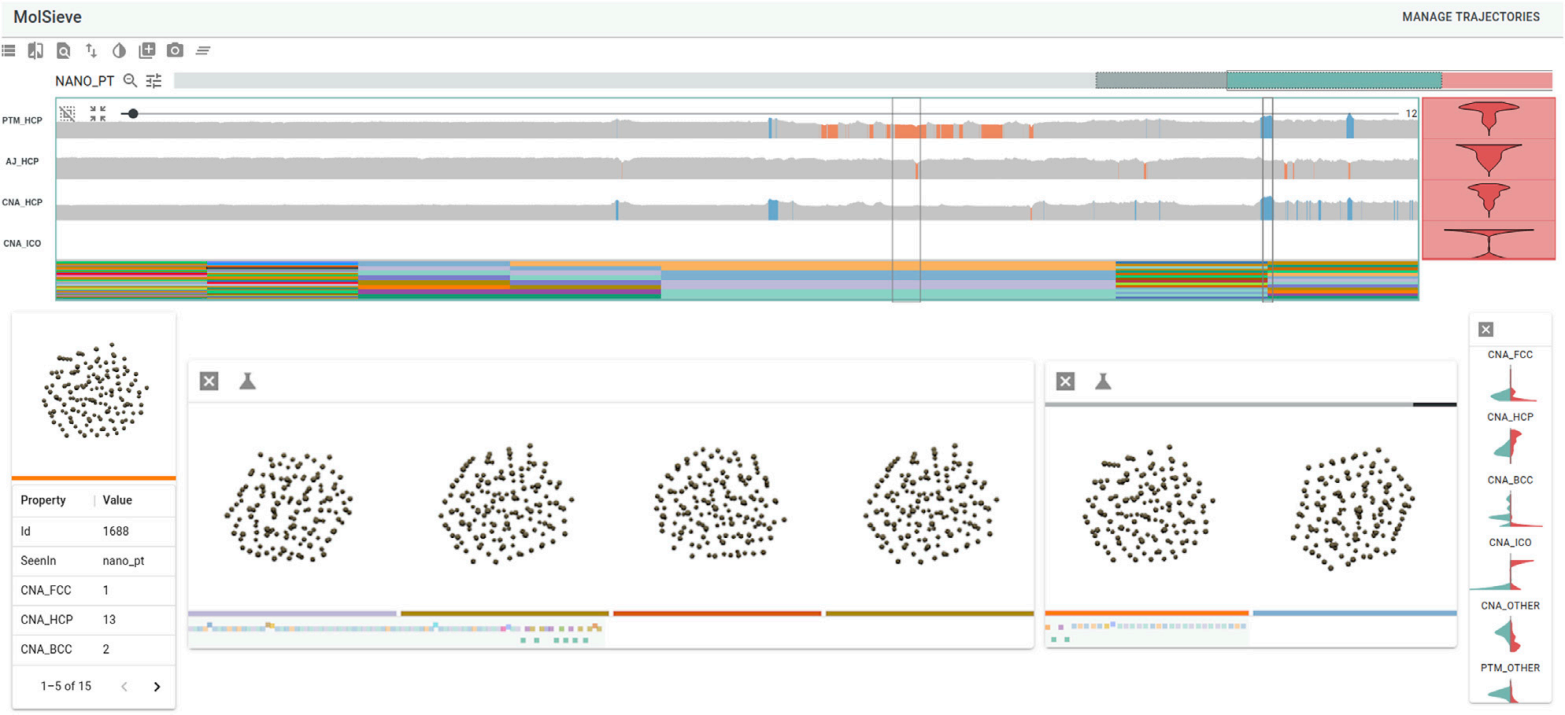
MolSieve is a progressive visual analytics system for comparing multiple long-duration simulations. It is able to quickly identify and compare regions of interest within immense simulation ensembles thanks to its combination of control charts, data-reduction techniques and highly informative visual components (Hnatyshyn et al., 2024). We thank the authors for providing an image depicting a typical MolSieve session. Figure courtesy by Rostyslav Hnatyshyn and colleagues.

As mentioned in the introduction, scaling up visualization and analysis to handling ensembles of simulations is an important challenge that the community is slowly beginning to address. The simultaneous analysis and visualization of an ensemble of molecular simulations appears today to be a crucial evolution to tackle future datasets and needs. Some examples of these efforts include (Dreher et al., 2014), which show a concurrent display and analysis of 5 molecular dynamics trajectories of an ion channel pore region undergoing dynamic dewetting. Also, (Juárez-Jiménez et al., 2020), developed a framework for integrating ensemble molecular dynamics simulations interactively with virtual reality. Users can generate realistic millisecond-scale representations of conformational changes in proteins. Kocincová et al., 2017 introduce a visualization approach that addresses the divide between commonly used 1D and 3D representations. By incorporating details about the mutual positions of protein chains into the 1D sequential representation, users can observe spatial differences between proteins without the typical occlusion found in a 3D view. This representation enables the comparison of multiple proteins or a set of time steps in MD simulations. Such comparative visualization can be adapted for an ensemble of molecular simulations. Another visual analytics system, MolSieve (Hnatyshyn et al., 2024), enables the comparison of multiple long-duration simulations modeled by discrete Markov chains (Figure 13). It reduces molecular dynamics simulations to their essential components (super-states and transition regions) to facilitate their analysis and comparison. The MolSieve interface allows, among other things, adjusting the exploratory parameters of each trajectory and provides a widget for comparing sub-sequences. In the same way in which multiscale molecular dynamics simulations have gained in popularity (Dans et al., 2016; Dommer et al., 2023; van Keulen et al., 2023), analysis and visualization tools for these simulation ensembles must evolve accordingly. To tackle this task one may take inspiration on existing databases of molecular simulations that comprise visualization. We present below a non-comprehensive list of existing databases. In addition, we discuss efforts that address sharing of molecular dynamics data.

### 5.1 Databases for molecular simulations

In 2003, BioSimGrid (Wu et al., 2003), a distributed database for biomolecular simulations was developed. It provides data on the conformational dynamics and energetics of complex biomolecular systems. BioSimGrid comes up with essential analysis tools such as root mean square deviation and fluctuation, average structure, internal angles, interatomic distances, molecular volumes, surface areas, and other geometrical properties. These tools empower users to conduct a variety of studies on the trajectories. Since 2004, the Ascona B-DNA (ABC) consortium has aimed at the systematic characterization of the physical properties of DNA nucleotide sequences by building an extensive database of trajectory ensembles (Beveridge et al., 2004). The effort continues within the ABCix project that has extended the sequence length in 2021 as described at https://mmb.irbbarcelona.org/webdev/slim/ABC/public/. In 2004 as well, the ProMode (Wako et al., 2004) database was established, containing normal mode analyses on protein molecules with a full-atom model. It allows the visualization of dynamic domains and their interrelated screw motions as determined by the Normal Mode Analysis (NMA) results. Additionally, graphical representations are provided for the properties of each normal mode vibration and their time averages, including fluctuations in atom positions, variations in dihedral angles, and correlations between atomic motions. These graphics serve to characterize collective motions in greater detail.

In 2005, Yang et al. calculated the dynamics using the Gaussian Network Model for 20,058 structures sourced from the Protein Data Bank (Burley et al., 2022), providing details on the equilibrium dynamics at the level of individual residues. Then, they stored these results on a web-based system named iGNM (Yang et al., 2005). Accessible on the platform are static and animated images that depict the conformational mobility of proteins across a wide range of normal modes.

In 2008, the CGDB (Chetwynd et al., 2008) database was created, focusing on membrane protein/lipid interactions through coarse-grained MD simulations. The database includes predictions regarding the positioning of membrane proteins within the lipid bilayer. In 2010, van der Kamp et al., 2010 developed Dynameomics, a database with over 7000 simulations of more than 1000 proteins totaling ∼200 μ s. It contains a range of proteins that represent almost all globular protein domains. Dynameomics can be exploited to derive both broad and detailed insights into the dynamics and folding/unfolding processes of proteins, pertinent subsets of proteins, and individual proteins. Several applications can be considered, including protein folding, mutation effects and drug design. In the same year, a database of atomistic molecular dynamics trajectories, named MoDEL (Meyer et al., 2010), emerged with over 1700 protein trajectories representative of monomeric soluble structures in the protein data bank (PDB). MoDEL is structured into five blocks. The initial block facilitates the automatic setup of molecular dynamics (MD) simulations, the second block enables error detection and trajectory validation, the third functions as a data warehouse, the fourth offers analysis tools, and the final block comprises a web server with its corresponding web applications. It is versatile and applicable to a wide range of objectives, covering evolutionary studies, biophysical analysis and drug design processes.

In 2015, the MemProtMD database (Stansfeld et al., 2015) was built on previous concepts initially developed around the CGDB (Chetwynd et al., 2008). It comprises a simulation pipeline designed to forecast the positioning of a membrane protein within a lipid bilayer. The pipeline encompasses a protocol for recognizing new membrane protein structures in the Protein Data Bank (PDB) and the analysis of lipid binding sites, as well as local bilayer deformation caused by membrane proteins. In 2019, in order to share this data with the scientific community, the MemProtMD web database (Newport et al., 2019) was established as a repository for structures of membrane-embedded proteins and their interactions with lipids. Simulations and the outcomes of subsequent analyses are accessible via a web browser, allowing 2D visualizations of membrane protein topology and depicting lipid contact data. In addition, it offers interactive 3D visualizations of the assembled bilayer.

In 2016, the BIGNASim (Hospital et al., 2016) database was developed as a portal of structure and analysis specifically designed for nucleic acids simulation data. The available analyses tools encompass backbone geometries, helical analysis, NMR observables, and various mechanical analyses. Both individual trajectories and combined meta-trajectories are included.

In 2018, a repository of G protein-coupled receptor (GPCR) data, named GPCRdb (Pándy-Szekeres et al., 2018), was constructed. It provides reference data, web tools and diagrams designed for a diverse audience across multiple disciplines, studying GPCR function, drug design or development. The tool works directly in the web browser, enabling rapid analysis of structures. In 2020, a collaborative initiative resulted in the establishment of an open, interactive, and standardized database for GPCR MD simulations (Rodríguez-Espigares et al., 2020a; Rodríguez-Espigares et al., 2020b). This web platform incorporates visualization features along with a comprehensive analysis toolbox, allowing users to visualize, analyze, and share GPCR molecular dynamics data.

### 5.2 Molecular dynamics data sharing

These efforts go hand in hand with sharing of molecular dynamics data (Abraham et al., 2019; Hildebrand et al., 2019; Hospital et al., 2020), for which protocols have been designed (Tiemann et al., 2017; Pacheco et al., 2019; Amaro and Mulholland, 2020; Kampfrath et al., 2022; Tiemann et al., 2023). Indeed, in 2017, (Tiemann et al., 2017), developed MDsrv, a tool designed to visualize interactively MD trajectories within web browsers, without the need for specialized knowledge in MD software. In 2022, this tool was enhanced by (Kampfrath et al., 2022) to simplify the process of uploading and sharing MD trajectories, while also improving their online streaming and analysis capacity. In 2023, indexed approximately 250,000 files and 2,000 datasets from Zenodo, Figshare and Open Science Framework (Tiemann et al., 2023; Pacheco et al., 2019) developed PCAViz, a freely available toolkit designed for sharing and displaying MD trajectories directly through web browsers. PCAViz consists of two main components: the PCAViz Compressor, responsible for compressing and storing simulation data efficiently, and the PCAViz Interpreter, responsible for decompressing the data within users’ web browsers and seamlessly integrating it with various browser-based molecular visualization libraries such as 3Dmol.js, NGL Viewer, and others. Still in the context of sharing data, (Riccardi et al., 2019), addressed an important issue in research communication, highlighting the challenges related to the reproducibility and transferability of research results across different groups. They examined this matter from the standpoint of computational biophysics and biochemistry. Thus, they propose to develop a platform dedicated to bring three elements together: the data, the users and the institutions. Recently, in the context of the global pandemic, the international community, as acknowledged in (Amaro and Mulholland, 2020), realized the necessity to adapt conventional practices to enhance the overall effectiveness of the global response to the pandemic. The research teams involved used preprint servers such as arXiv, bioRxiv, and ChemRxiv and open access data repositories such as Zenodo (Zenodo, 2013) to make their research available as quickly as possible. They also made models and trajectories available through open data sharing platforms. They commited to applying reflective, permissive (and open source) licensing strategies and to sharing algorithms and methods in appropriate repositories such as GitHub (Github, 2023).

In this section, we highlighted efforts that begin to address the visualisation of ensemble MD simulations. In addition, we presented examples of databases and data sharing approaches for molecular dynamics, which go hand-in-hand with the visualisation efforts. In the next section, we discuss common tools and software in this area.

## 6 MD-visualization software and tools

Visualizing complex molecular dynamics trajectories is a key challenge of this decade, which is becoming more and more accentuated with the evolution of MD simulations. MD visualization tools vary in their capacity to visualize and analyze trajectories of complex molecular systems. The ability to handle complex systems depends on factors such as the tool’s rendering capabilities, performance optimizations, analysis features, and support for different molecular formats. We will first describe the features one can generally expect from MD visualization tools, standalone systems and/or web-platforms when it comes to handling complex molecular systems. One criteria to include a given tool in this section was it’s ability to manage common trajectory file formats and simulation systems.

### 6.1 Standalone software

Among standalone systems, we selected VMD (Visual Molecular Dynamics) (Humphrey et al., 1996), UCSF ChimeraX (Goddard et al., 2018), YASARA (Krieger and Vriend, 2014) and CAVER Analyst (Kozlikova et al., 2014). VMD (Humphrey et al., 1996) stands as a versatile software tool, compatible across multiple platforms, designed for the simulation, visualization, and analysis of extensive biomolecular systems. It is renowned for its strong capacity to visualize and analyze trajectories of complex molecular systems. It can handle biomolecular complexes, macromolecular assemblies, and intricate molecular structures. The tool offers advanced rendering options (Stone et al., 2016a), efficient handling of large systems, and support for various file formats. VMD’s scripting capabilities also enable users to create custom analysis workflows for complex systems. To increase the efficiency of graphics rendering for large molecular systems, (Stone et al., 2016b), adapted VMD to support the Embedded-system Graphics Library (EGL). This approach allows parallel visualization, without relying on windowing system software, and effectively rendering supporting multi-GPU compute node configurations.

Skånberg and colleagues (2018) introduced VIA-MD, a visual exploration environment designed for large-scale spatio-temporal molecular dynamics simulation data. This tool provides a linked interactive 3D exploration that combines geometry with statistical analysis through dynamic temporal windowing and animation. By employing semantic-level descriptions and hierarchical aggregation of molecular properties, VIA-MD enables interactive filtering, allowing users to effectively identify spatial, temporal, and statistical patterns (see Section 4.4.2).

UCSF ChimeraX (Goddard et al., 2018) is an updated version of the Chimera (Pettersen et al., 2004) molecular graphics program, with an expanded feature set and improved performance. ChimeraX is designed to handle complex molecular systems, making it suitable for visualizing and analyzing macromolecular assemblies, membrane systems, and integrative models. The tool offers various rendering styles, support for cryo-electron microscopy data, and interactive exploration of complex structures. ChimeraX’s focus on integrative visualization can aid in understanding complex molecular interactions.

**FIGURE 14:**
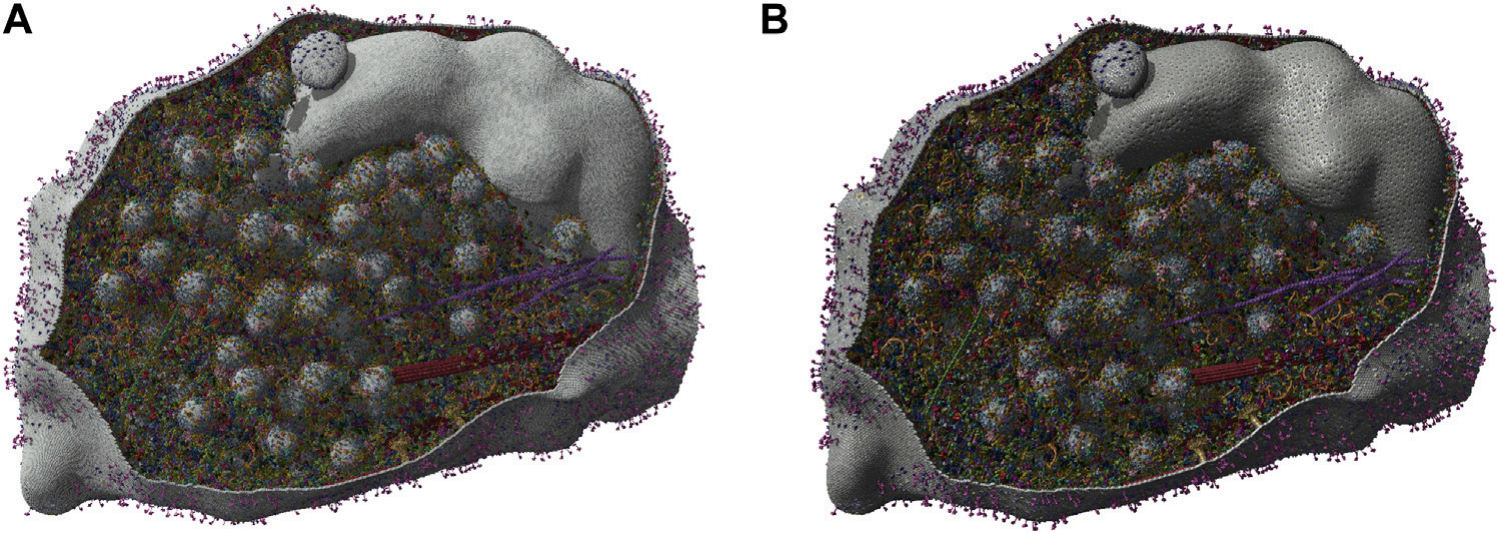
Visualization of *presynaptic* bouton with Yasara Viewer using Vulkan Graphics API (Ozvoldik et al., 2021). **(A)** Cut open coarse-grained *presynaptic* bouton pet model. **(B)** Cut open all-atom *presynaptic* bouton model. Image generated with YASARA Viewer (Krieger and Vriend, 2014).

YASARA (Krieger and Vriend, 2014) is a molecular graphics and modeling software that supports the visualization of large and complex molecular systems, including simulations. Indeed, for the efficient interactive visualization of gigastructures YASARA employs a grid-based algorithm (Ozvoldik et al., 2021) to create such levels of detail (LODs). As a result, the user can interactively visualize giant models such as the *presynaptic* bouton with 3.6 billion atoms (see Figure 14). YASARA employs two methods for data compression: Assembly involves using coarse-grained “pet molecules,” leading to a 50-fold reduction in atom count (see Figure 14A), while all-atom visualization utilizes GPU instancing, resulting in memory requirements reduced by a factor of 40–1000 (Figure 14B).

CAVER Analyst (Kozlikova et al., 2014) is a valuable tool for researchers studying the accessibility and dynamics of pathways within protein structures. It excels at visualizing and analyzing tunnels, channels, and cavities in complex molecular systems obtained from MD simulations. Its focus on pathway analysis and its ability to provide insights into potential binding sites make it particularly useful in drug discovery and structural biology research.

In summary, these software packages stand out as powerful tools for visualizing and analyzing trajectories of complex molecular systems. They offer diverse rendering options, analysis tools, and support for large assemblies. Many of these software tools can be customized and tailored to specific requirements and workflows, providing great flexibility for complex tasks. However, such platforms have their drawbacks, including installation requirements and setup procedures on a local computer, making them less accessible for remote collaboration. They may have a complex interface and require training to use effectively.

### 6.2 Web-based molecular graphics

Advances in web technology allowed the emergence of web-based molecular graphics that provide visualization and analysis of trajectories of complex molecular systems. The use of local rendering solutions is more common than a video stream from a remote rendering server. In the following paragraphs, some notable options are presented.

The LiteMol suite (Sehnal et al., 2017) is an interactive web-platform, designed for the visualization of extensive macromolecular structure data, offering users an engaging and responsive experience across all contemporary web browsers and mobile devices. It supports multiple file formats and provides tools for exploring and analyzing complex molecular systems, including trajectories. It integrates data delivery services, a compression format and a molecular viewer (LiteMol Viewer (Sehnal, 2016; Sehnal et al., 2020). The LiteMol suite is freely available at https://litemol.org/.

NGL Viewer (Rose and Hildebrand, 2015) is a highly interactive WebGL-based molecular visualization tool. It can handle complex structures and trajectories, allowing users to visualize and analyze molecular dynamics simulations directly in their web browsers. (Rose et al., 2018). enhanced the NGL viewer ability to use memory resources effectively and minimize unnecessary memory consumption. These empower NGL to dynamically download and visualize molecular complexes containing millions of atoms on desktop computers and smartphones. The NGL Viewer is open source and can be freely accessed at https://nglviewer.org/.

**FIGURE 15:**
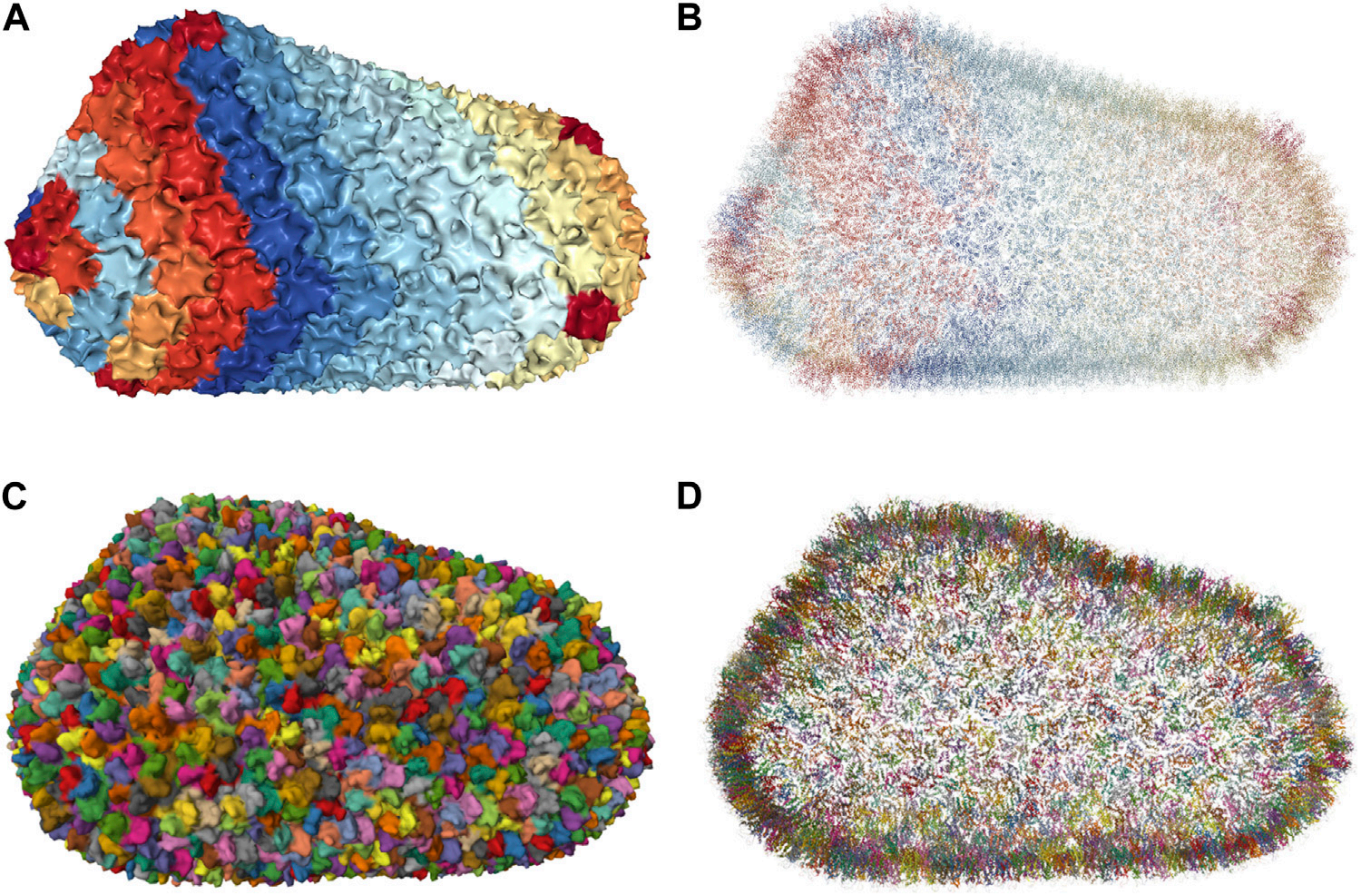
Atomic-level visualization of the entire HIV-1 capsid (PDB ID: 3J3Q) with molecular web viewers. **(A)** Surface representation of the HIV-1 capsid using NGL Viewer. **(B)** Cartoon representation of the HIV-1 capsid using NGL Viewer. **(C)** Surface representation of the HIV-1 capsid using Mol* Viewer. **(D)** Cartoon representation of the HIV-1 capsid using Mol* Viewer (Rose and Hildebrand, 2015; Sehnal et al., 2021).

Mol* Viewer (Sehnal et al., 2021) is another WebGL-based molecular visualization tool that inherits many features from the LiteMol suite (Sehnal et al., 2017) and the NGL Viewer (Rose and Hildebrand, 2015). It enables the simultaneous visualization of hundreds of (superimposed) protein structures, the streaming of MD simulation trajectories, or the display of huge structures and assemblies built using integrative/hybrid methods. Mol* Viewer is open source and freely available at https://molstar.org/. Figure 15 shows the visualization of the HIV-1 capsid structure reported by Zhao et al., 2013 with 2.440.800 atoms in both NGL and Mol* Viewers using surface and cartoon representation.

Web viewers can be accessed from any device with an internet connection, simplifying collaboration and sharing of results. They usually have a simple and user-friendly interface, making it easier for non-experts to use the software. These tools can be updated easily and regularly, allowing for new features and bug fixes to be released faster. They in turn have some disadvantages related to performance limitations due to the computational requirements of molecular dynamics simulations. Functionality limitations compared to standalone applications may arise for more complex or specialized tasks and handling of large files. In addition, the web-based and distributed nature may bring up security concerns as these systems may store and process sensitive data on remote servers. Not to mention the cost of hosting and storing, this factor is a major design constraint for any web-based software and in particular for molecular dynamics data.

### 6.3 VR tools

VR molecular visualization is a niche application in comparison to the very broadly used desktop visualization, particularly in terms of available mature software solutions and frequency of resorting to such approaches in the current research landscape. For exploring molecular trajectories in virtual reality, only few choices are available and visualization features are more restricted in comparison to the above-mentioned stand-alone and web-based viewers. UnityMol (Laureanti et al., 2020) provides features for trajectory playback and interactive MD simulations in VR. Narupa is another VR-focused environment, designed with interactive MD in mind (O’Connor et al., 2019). Other software packages may offer some trajectory support. An online list of Molecular Visualization XR (eXtended Reality) software is available through https://github.com/davous267/molecular-visualization-in-virtual-environments/. A recent review (Kuta et al., 2023) discusses molecular visualization in immersive virtual environments as a whole, with a section dedicated to molecular dynamics simulations.

In conclusion, the choice between web viewers and standalone applications for molecular visualization software depends on the specific requirements and goals of the project, as well as the level of expertise of the users. Virtual reality exploration is still a niche application in terms of broad adoption.

## 7 MD-vis challenges and perspectives

In this paper, we discussed the impressive progress in the field of molecular dynamics simulations as well as the existing visualization tools. In addition, we presented the visual abstraction formalism to help classify the efforts and guide the discussion. Based on this analysis, we were able to identify a series of challenges that we present in this section:

• The increasing complexity in data visualization.
• Extending the repertoire of representations (image axis).
• The lack of standards for visualisation.
• Addressing visualisation of ensembles of MD trajectories.
• Technical challenges for efficient graphical rendering.
• The gap between advances in computer graphics and bioinformatics.
• The limitations of VR/AR/MR tools on MD visualisation.

### 7.1 The increasing complexity in data visualisation

The challenge of the increasing data complexity does not lie solely in the ability to visualize them. This visual complexity makes it difficult to understand and interpret the data. Indeed, multiscale design substantially reduces overall complexity by employing diverse data representations to emphasize noteworthy features. Nonetheless, the existence of numerous representations for the same data can impose a cognitive burden on the observer when visualizing the data trying to mentally relate the different representations. In this sense, the visualization task requires a visualization strategy to promote the pertinent view point, highlighting the important visual information, and streamlining the representation to ensure intuitive perception of information without sacrificing its completeness. We believe that this is one of the greatest challenges of scientific visualization in the present context. One interesting perspective could be to make use of the new technologies to remove some information from the visual channel - if possible - and transfer it to another modality such as audio for instance (Rau et al., 2015; Ballweg et al., 2016; Arbon et al., 2018), to reduce the amount of remaining information on the visual channel. This reduced visual channel information will be better understood and the cognitive load will be more manageable. Nevertheless, it is interesting to consider how the new generation of graphical representations may capture MD features in a static image. This ties to the next challenge for future graphical representations.

### 7.2 Extending the repertoire of representations

The challenge of extending the repertoire of representations for visualizing trajectory data is often mentioned in the literature since 2005 (Goodsell, 2005). Improving the visualization of MD trajectories of complex systems remains a relevant challenge, because the simulations and the visualization of these data do not evolve at the same rate. Indeed, we have observed that the existing visualization techniques do not fully meet the needs of molecular dynamics. Frame-by-frame visualization tools were implemented based on classic representations. Also, levels of abstraction or multiscale representations were proposed to allow the visualization of complex data. However, the complexity of the generated data is increasingly intertwined over long simulation times. The existing graphic representations date from the 90 s and multiscale representations may no longer be sufficient, especially with the interactions and transformations that may occur during molecular dynamics. Today, the challenge is to design a new generation of graphic representations dedicated to molecular dynamics. These representations should keep traces of the dynamics as the trajectory is read. For example, representations which, at time t of the trajectory, would allow the user to know what occurred in previous frames could be very helpful for the modeler to analyze and understand the biomolecule’s behaviour. Thinking about representations for dynamics by considering the complexity of the data will make it possible to offer more adequate tools for the visualization of these trajectories representing physical phenomena in greater detail and enhancing the perceptual and cognitive effectiveness of visual representations.

### 7.3 Addressing visualisation of ensembles of MD trajectories

In the same context of data complexity, the simultaneous analysis and visualization of a set of molecular simulations is another challenge, which has not been extensively addressed in the literature. Today, it seems that addressing this challenge will be a crucial development to handle upcoming datasets and requirements. Comparative visualization becomes imperative in this context, facilitating the comprehension of relationships and patterns across numerous simulations. Similarly, as multiscale molecular dynamics simulations gain popularity, there is a pressing need for the corresponding evolution of analysis and visualization tools tailored to these simulations. One possible road-map that has not been explored so far, is to connect visualisation tools with AI-based companions, that could be trained to handle complex selection queries involving multiple trajectory data, and provide both analysis and interactive visualisation.

### 7.4 Technical challenges for efficient graphical rendering

Efficient graphical rendering requires massive computational resources for large molecular systems. When dealing with larger and more complex macromolecular structures, novel visualization challenges arise in both rendering approaches and memory management. The size of these structures may exceed the capacity of GPU memory, and the data streaming from CPU memory could be insufficient for real-time applications. The use of streaming through pipelines from powerful machines is a solution that could be considered to visualize large scale simulations. Parallel rendering (or distributed rendering) is another eventual solution to render frames faster. This makes it possible to manage the complexity of the data and to achieve a high graphics rendering quality, but it requires a powerful graphics server. Whereas modelers already use HPC to generate the molecular dynamics simulations, the access to these supercomputers is not unlimited. Thus, it will be suitable to limit the use of such equipment for visualizing MD simulations. *In-situ* approaches can be considered. They present a potential for optimizing the use of supercomputers. Indeed, they take advantage of “dead times” on the supercomputer during the simulation to prepare visualizations and the analysis/reporting at the end of the simulation (Vishwanath et al., 2011; Dreher and Raffin, 2014; Malakar et al., 2015; Dorier et al., 2016; Dirand et al., 2018).

### 7.5 The gap between advances in computer graphics and bioinformatics

Another challenge that comes up frequently in many reviews since 2011 and remains relevant is the gap between computer science evolution and bioinformatics (Martinez et al., 2019b). Computer scientists develop new concepts and graphical tools that improve graphic rendering and reduce execution time. However, these programs are often proof-of-concept demonstrators and may be dedicated to other areas such as computer games. On the one hand, bioinformaticians will not necessarily be aware of these novelties and they may not have the computer graphics background to readapt these solutions. To overcome this problem, it is important to create collaborations between both communities. Participating at scientific events bringing together structural biologists and researchers working on computer graphics could be a good way to create collaborations that are beneficial for researchers in both domains working in the field of molecular visualization. Expanding the education of bioinformaticians on the computer graphics concept and tools can allow readapting the graphics developments from different fields of application. Even if a novel and advantageous rendering algorithm is found and made available, turning it into a supported and sustainable tool poses an additional challenge.

### 7.6 The limitations of VR/AR/MR tools for MD visualization

As briefly mentioned in the software discussion, Section 6, the accessibility of VR headsets and the availability of VR packages on several MD simulation visualization softwares, permit the use of this technology to explore biological phenomena. Nevertheless, it is necessary that the existing software packages offer new navigation and 3D interaction tools adapted to the context of molecular dynamics to facilitate the manipulation of the molecular data respecting their physical and chemical properties. The major problem that could arise with the use of this technology is the size and complexity of the MD trajectory data being visualized. Many VR systems have limitations in terms of processing power, storage capacity, and display resolution, which impacts the quality and detail of the visualization. Additionally, the ability to interact with and manipulate the data in real-time is usually limited by the VR system’s capability. To overcome these limitations, it may be necessary to use specialized VR systems that are optimized for scientific visualization and research and may for instance leverage cloud-based streaming approaches.

AR is currently less successful than VR in this field and represents an untapped potential. Yet AR is a good alternative for people who are not at ease with total immersion in a virtual environment, but it does not always offer 3D perception. However, in modeling, the important aspect is the perception of the 3D structure, so Mixed Reality (MR) should be a key technology for the future. It offers a 3D perception of the structure while maintaining contact with the real world. Combined with Spatial Computing, the ability of devices to be aware of their surroundings and to represent them digitally opens immense possibilities. These techniques will enable much-needed new avenues for visualization and interaction. However, they will face the same problem as VR in terms of data complexity. Finally, collaborative work interfaces should be more developed to allow coworkers to explore the same data from various viewpoints. This technology offers a new approach to explore MD simulations, for instance by visualizing the data from different perspectives thus reducing the problem of occlusion. In addition, VR, MR and SC technologies combined with the collaborative work can be a powerful tool for the dissemination of knowledge.

## 8 Conclusion

This report highlights scientific and technological advances in the visualization for complex molecular dynamics simulations. We acknowledge the potential of multiscale molecular representations, visual abstraction, and aggregation for a better understanding of molecular behavior and interactions, but also emphasize the urgent need for further exploration. Despite some new representations tailored to MD visualization, the field remains largely unexplored, especially with regard to the increasing dimensions of simulated systems that exceed conventional visualization capabilities.

There is a need to re-evaluate existing representations and visualization techniques to identify and respond to the shortcomings they present in light of advancements in MD simulations. This includes not only the refinement of current methods, but also the development of new visualization metaphors for MD simulations. These new representations should aim to simplify both temporal and spatial scale complexity to reduce the cognitive burden and facilitate the identification of significant events within the simulations. Furthermore, it is important to bridge the gap between the scientific visualization of raw simulation data and the analytical visualization. By linking the scientific visualization of the raw simulation data with the visualization of the analysis data, we hope to effectively navigate the complexity of molecular dynamics and open up new areas of understanding.
